# Library Screening, In Vivo Confirmation, and Structural and Bioinformatic Analysis of Pentapeptide Sequences as Substrates for Protein Farnesyltransferase

**DOI:** 10.3390/ijms25105324

**Published:** 2024-05-13

**Authors:** Garrett L. Schey, Emily R. Hildebrandt, You Wang, Safwan Diwan, Holly A. Passetti, Gavin W. Potts, Andrea M. Sprague-Getsy, Ethan R. Leoni, Taylor S. Kuebler, Yuk Y. Sham, James L. Hougland, Lorena S. Beese, Walter K. Schmidt, Mark D. Distefano

**Affiliations:** 1 Department of Medicinal Chemistry, University of Minnesota, Minneapolis, MN 55455, USA; schey013@umn.edu; 2 Department of Biochemistry and Molecular Biology, University of Georgia, Athens, GA 30602, USA; erh@uga.edu (E.R.H.); erl99598@uga.edu (E.R.L.); wschmidt@uga.edu (W.K.S.); 3 Department of Biochemistry, Duke University School of Medicine, Durham, NC 27710, USA; you.wang@duke.edu (Y.W.); lorena.beese@duke.edu (L.S.B.); 4 Department of Chemistry, University of Minnesota, Minneapolis, MN 55455, USA; diwan005@umn.edu (S.D.); hap5160@psu.edu (H.A.P.); gwp002@morningside.edu (G.W.P.); 5 Department of Chemistry, Syracuse University, Syracuse, NY 13244, USA; anspragu@syr.edu (A.M.S.-G.); hougland@syr.edu (J.L.H.); 6 Bioinformatics and Computational Biology Graduate Program, University of Minnesota, Minneapolis, MN 55455, USA; kuebl010@umn.edu (T.S.K.); shamx002@umn.edu (Y.Y.S.); 7 Department of Integrative Biology and Physiology, University of Minnesota, Minneapolis, MN 55455, USA; 8 Department of Biology, Syracuse University, Syracuse, NY 13244, USA; 9 BioInspired Syracuse, Syracuse University, Syracuse, NY 13244, USA

**Keywords:** enzymology, farnesyltransferase, peptide libraries, protein prenylation

## Abstract

Protein farnesylation is a post-translational modification where a 15-carbon farnesyl isoprenoid is appended to the C-terminal end of a protein by farnesyltransferase (FTase). This process often causes proteins to associate with the membrane and participate in signal transduction pathways. The most common substrates of FTase are proteins that have C-terminal tetrapeptide CaaX box sequences where the cysteine is the site of modification. However, recent work has shown that five amino acid sequences can also be recognized, including the pentapeptides CMIIM and CSLMQ. In this work, peptide libraries were initially used to systematically vary the residues in those two parental sequences using an assay based on Matrix Assisted Laser Desorption Ionization–Mass Spectrometry (MALDI-MS). In addition, 192 pentapeptide sequences from the human proteome were screened using that assay to discover additional extended CaaaX-box motifs. Selected hits from that screening effort were rescreened using an in vivo yeast reporter protein assay. The X-ray crystal structure of CMIIM bound to FTase was also solved, showing that the C-terminal tripeptide of that sequence interacted with the enzyme in a similar manner as the C-terminal tripeptide of CVVM, suggesting that the tripeptide comprises a common structural element for substrate recognition in both tetrapeptide and pentapeptide sequences. Molecular dynamics simulation of CMIIM bound to FTase further shed light on the molecular interactions involved, showing that a putative catalytically competent Zn(II)-thiolate species was able to form. Bioinformatic predictions of tetrapeptide (CaaX-box) reactivity correlated well with the reactivity of pentapeptides obtained from in vivo analysis, reinforcing the importance of the C-terminal tripeptide motif. This analysis provides a structural framework for understanding the reactivity of extended CaaaX-box motifs and a method that may be useful for predicting the reactivity of additional FTase substrates bearing CaaaX-box sequences.

## 1. Introduction

**Figure 1:**
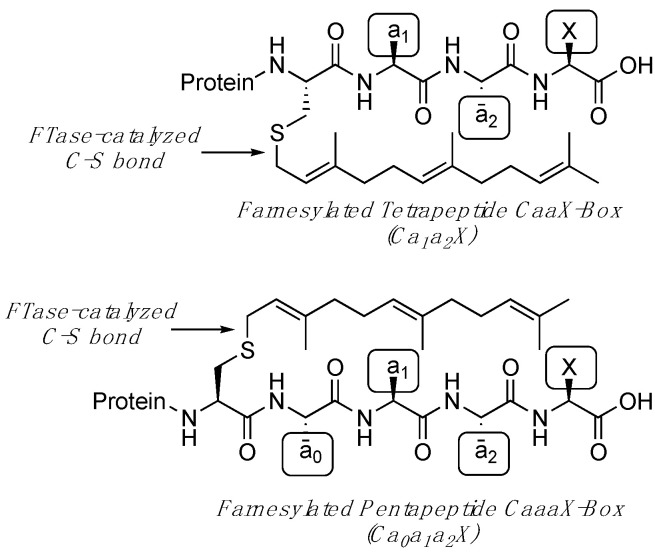
Structure and nomenclature for farnesylated tetrapeptide and pentapeptide sequences. For tetrapeptide sequences, the C-terminal position is denoted by X, the penultimate residue by a_2_, and the next residue on the N-terminal side as a_1_. For pentapeptide sequences, this nomenclature has been retained for consistency and clarity. Thus, the fourth residue from the C-terminus is denoted a_0_.

Protein prenylation is a post-translational modification in which a hydrophobic isoprenoid group is covalently attached to the thiol side chain of a cysteine residue located near the C-terminus of a protein via enzymatic formation of a new C-S bond (Figure 1). Farnesyltransferase (FTase) transfers a 15-carbon farnesyl group using farnesyl diphosphate (FPP) while Geranylgeranyltransferase-I (GGTase-I) employs a 20-carbon geranylgeranyl diphosphate (GGPP) prenyl donor [1]. These enzymes recognize proteins with a C-terminal tetrapeptide consensus sequence known as a “CaaX box”. In the canonical view of prenyltransferase selectivity, “C” is the cysteine residue that is covalently modified, “a” is usually an aliphatic amino acid, and the “X” is a residue that is largely responsible for determining whether the protein substrate is targeted by FTase or GGTase-I [2]. Protein prenylation is essential for proper cellular localization, protein–protein interactions, and signaling activity, and misregulation of prenylation is implicated in many diseases including cancer [3,4,5,6,7,8]. In addition, prenylation and the prenylation pathway have drawn considerable attention as potential targets for the treatment of Alzheimer’s disease, Hutchinson−Gilford progeria syndrome, and numerous other diseases [9,10,11,12,13,14]. In 2020, the first FDA-approved inhibitor of FTase, lonafarnib, initially designed as a potential cancer drug, was approved for the treatment of progeria since it prevents the prenylation of prelamin A [15,16]. Another inhibitor, Tipifarnib, has long been under investigation for cancer therapy and is still under investigation in clinical trials for head and neck squamous cell cancers driven by H-RAS mutations [17,18]. Inhibitors that target enzymes involved in the processing and modification of prenylated proteins are also under investigation [19,20,21].

FTase manifests broad substrate specificity, catalyzing the transfer of a farnesyl group from FPP to a wide variety of protein and peptide substrates, and many attempts have been made to define what amino acids are allowed or not allowed in the CaaX sequence [22,23,24]. Interestingly, it has been found that while CaaX binding is largely controlled by the X amino acid, there is synergy between the amino acids that affects their efficiency as substrates [25,26,27]. The flexibility of the peptide binding site has been used in the rational design of novel mutant FTases for orthogonal peptide reactivity and to enhance the ability to accept fluorescent isoprenoid analogs [28,29,30]. While the canonical model of the CaaX-box is generally well understood, it has recently been found that certain non-canonical length sequences other than the four-residue CaaX motif can also be farnesylated by both yeast and mammalian FTase orthologs, specifically both tripeptides and pentapeptides [31,32]. The prenylation of pentapeptide CaaaX sequences was first observed in yeast, and the initial evaluation of CaaaX substrate space found the sequence CMIIM to be the most efficiently prenylated of those evaluated. In vitro assays indicated that this peptide was a reasonable substrate, although approximately 10-fold less efficient compared with the most efficient CaaX peptides such as CVLS [31]. None of these peptides were found to be substrates for GGTase-I.

Due to the past three decades of research, the selectivity rules for CaaX-containing peptides have become increasingly well defined. However, there are many questions remaining about how this information might apply to pentapeptide CaaaX sequences since it is known that the FTase enzymes from different organisms display different preferences for peptide substrates [33]. Peptide libraries are particularly useful tools for the interrogation of enzyme specificity [34,35]. Previous work in our lab has relied on the analysis of peptide libraries through the use of an alkyne-containing isoprenoid analog to allow for biotin attachment by derivatizing with biotin–azide via copper-catalyzed azide−alkyne cycloaddition [33]. Since that approach relies on synthetic isoprenoid analogs that may perturb enzyme specificity, it may provide misleading results concerning the specificity with the native substrate FPP [36]. In an effort to study peptide libraries with the native FPP substrate, we recently developed a MALDI-MS-based detection assay. Peptide libraries were synthesized with a Dansyl-Glycine (DsG) and RAG sequence upstream of the variable CaaaX sequence to aid in solubility and ionization [37]. This method relied on the 204 Dalton mass shift due to the addition of the farnesyl group to easily observe the formation of prenylated product peptides. The gentle ionization of MALDI resulted in clear results by producing singly charged ions without fragmentation, making it highly amenable for the analysis of peptide libraries [38,39].

We utilized this peptide library approach to study variations of the most efficiently prenylated CaaaX sequence, CMIIM, using yeast FTase (yFTase). Over 30 new prenylated sequences were observed using that approach and validation of eight of these sequences in a yeast-based reporter protein assay showed that they were prenylated in vivo. In addition, a curated search of the human genome revealed that there may be many potentially prenylatable CaaaX sequences. Analysis of one such sequence, CSLMQ, suggested that it was prenylated as efficiently as the sequence CVLS (via HPLC), the CaaX box present at the C-terminus of H-Ras, and was also prenylated in yeast [37]. To expand our understanding of the recognition of CaaaX sequences here, we have analyzed CMIIM libraries with rat FTase (rFTase), as well as generated and tested a new set of libraries based on CSLMQ, screening with both yFTase and rFTase. In addition, we have looked more broadly by evaluating 192 CaaaX sequences that occur naturally in the human genome. After initially screening the above peptides using the MALDI-based method, 21 of the positive hits were further evaluated in a yeast-based human FTase reporter assay to determine their level of prenylation. Finally, X-ray crystallographic analysis of the structure of TKCMIIM bound to *Cryptococcus neoformans* FTase (CnFTase) in concert with molecular dynamics simulations and bioinformatic analysis allowed us to formulate a model for understanding and potentially predicting the prenylation efficiency of pentapeptide sequences.

## 2. Results

### 2.1. Identification of Novel Substrates from the CMIIM Motif Using MALDI Analysis

**Figure 2:**
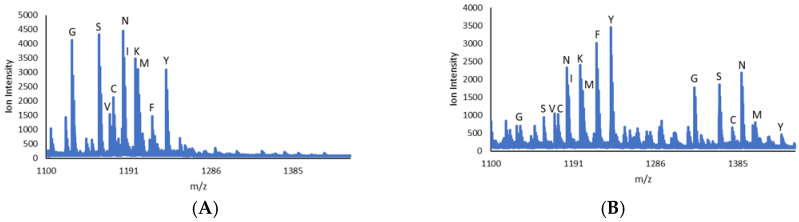
CMa_1_IM Library 1 (**A**) before and (**B**) after farnesylation with 2 µM rFTase at 35 °C for 8 h. The identity of the residue in the a1 position is indicated with the letter above each peak. a1 = C, G, M, N, S, and Y, with A, E, H, Q, R, and T shown in Figure S1.

Utilizing our previously described method of evaluating peptide libraries by MALDI-MS, libraries based on CMIIM were analyzed with rFTase as a comparison to previously reported yFTase library data [37]. Using 2 µM enzyme, it was possible to observe a number of hits for the library CMa_1_IM where a_1_ is the variable position. Positive hits were identified as peaks corresponding to the mass change (+204 Daltons) due to the addition of a farnesyl group, and having a S:N ratio of 12, as in our previously reported work. Positive hits in this position included Ala, Arg, **Asn**, Cys, **Gln**, Glu, **Gly**, **His**, Met, **Ser**, **Thr**, and **Tyr**, with those in bold being shared between libraries analyzed using rFTase or yFTase (Figure 2 and Figure S1); the yFTase data have been previously reported [37]. Of the shared hits, the data were normalized by dividing the ionization of the prenylated peak by the combined ionization of the parent ion and product (Table 1).

Differences were observed in the prenylation levels of the peptides present in these libraries, with some amino acids such as Gly being preferred by rFTase and His being preferred by yFTase. Amino acids such as Gln appear to be prenylated equally by both enzymes. While this quantitative analysis should be interpreted with caution due to differences in the ionization efficiency of the product versus the reactant, it can provide a useful metric to estimate the efficiency of substrates between different enzymes.

Interestingly, analysis of the other three positions in the CMIIM-based libraries yielded no prenylated peptides at this enzyme concentration. While the rat enzyme is generally more stringent in its substrate specificity than the yeast enzyme, this is surprising as it was possible to find hits at all positions using yFTase [33]. However, increasing the concentration of the enzyme to 10 µM rFTase afforded some prenylated peaks, although the peak intensities were still quite low compared to earlier results with the DsGRAGCMa_1_IM libraries (Table 2, Figures S2–S7). This is in contrast to libraries analyzed using yFTase, where increasing the concentration of the enzyme did not significantly increase the peak intensity. It is somewhat difficult to interpret these latter results since those experiments required higher enzyme concentrations, but it is clear that rFTase has the most flexibility for the a_2_ position. While canonical hits such as Gln, Met, and Ser were observed at the X position using 10 µM rFTase, it is interesting that none were observed at lower enzyme concentrations, as this is the residue considered most important for substrate binding [27]. Finally, it was surprising that screening of the a_0_ position yielded no hits at the higher enzyme concentration. In general, tetrapeptide sequences have shown a similar number of hits for the aliphatic positions (a_1_ and a_2_), and previous analysis showed this trend proved to also hold for pentapeptides when analyzed with yFTase (Table 3). Therefore, it is striking that substantial differences in reactivity between the aliphatic positions were observed when using rFTase (Table 4). While substrate inhibition is certainly a possibility, it is difficult to use this as the reasoning behind our results.

Next, an HPLC-based fluorescence assay was employed to validate several of the peptide hits obtained in the MALDI-based screening. This assay is based on the observation that peptides manifest a dramatic increase in retention time in reversed-phase chromatography upon prenylation and has been used extensively in the field [31,37,40]. Prenylation of peptides containing CMGIM, CMNIM, and CMSIM sequences were reacted for 45 min with 200 nM rFTase giving conversions of 50%, 41%, and 31%, respectively, (Figures S8–S10). This is consistent with the conversion levels previously found with yFTase, although nothing stood out as a more efficient substrate than CMIIM for rFTase. It is important to note that the prenylated pentapeptide sequences proved to be particularly insoluble and were not observable or gave variable results in the HPLC assay. To prevent column clogging, samples had to be centrifuged prior to injection, which likely provided another mechanism for product loss. Hence, reactions were followed by monitoring the decrease in the unprenylated form of the peptide. Importantly, as control samples were subjected to the same incubation time as the enzymatic reactions, we can rule out precipitation as the reason for the decrease in starting material.

### 2.2. Identification of Novel Substrates from the CSLMQ Motif Using MALDI Analysis

Due to previous results showing that CSLMQ appeared to be a more efficient substrate than CMIIM, we next explored libraries based on that sequence [37]. Initially, examination of the libraries using 1 µM yFTase showed different trends than were observed with the CMIIM libraries (Table 3). In the case of CMIIM-based libraries, they contained a variety of hits at all positions other than a_0_. In contrast, with CSLMQ-based libraries (Figures S11 and S12), we observed three hits at the a_0_, fourteen at the a_1_ position, six at the a_2_ position, and two in the X position. Thus, the most permissive position was the a_1_ position. Normally, bulky aromatic amino acids in the aliphatic positions are not favored, but Tyr and Phe have been consistently observed in our libraries, with even the conformationally restrained Pro appearing as a hit. The tighter selectivity at positions other than a_1_ is interesting, as it might be expected that the generally more flexible yFTase could reveal more hits [33].

Reacting CSLMQ libraries in the presence of 3 µM rFTase yielded results indicating a surprising number of hits at all positions except a_0_, which only showed Ile as a hit (Figures S13–S20, Table 4). Given that yFTase is more relaxed in its selectivity for tetrapeptide CaaX sequences compared with the rFTase, the greater number of hits obtained using the rat enzyme with these pentapeptide libraries is surprising [33]. Positive hits included a variety of canonical amino acids and more unusual aromatic residues, including Trp in the a_2_ position. The X position also showed hits for charged amino acids including Asp and Glu, which are typically associated with single turnover reactions, although that is unlikely here given the enzyme-to-peptide ratio employed and the large number of peptide hits observed in that library reaction [25]. As noted above for the CMIIM-based libraries, the insolubility of the prenylated product peptides from the CSLMQ-based libraries also complicated the kinetic analysis of those latter reactions. However, it was possible to observe the prenylated product derived from the parent sequence CSLMQ (see Figure S21). As was observed with other prenylated peptides, the lipidated form of that peptide eluted from the reversed-phase HPLC column with a longer retention time relative to the unmodified form.

### 2.3. CaaaX Hits in the Mammalian Genome

To identify additional pentapeptide sequences that might be FTase substrates, a search of the human genome was performed using the search motif Ca_0_a_1_a_2_X, where any amino acids were allowed in the a_0_, a_1_, a_2,_ and X positions, finding over 1000 potential substrate sequences, as previously described [31]. From that list, 192 of those peptides were chosen (Table S1) by excluding sequences that had multiple charged residues, multiple conformationally challenging residues (Gly and Pro), or one of each from the aforementioned categories. This parallel synthesis effort employed an Intavis Multipep RS instrument, allowing for the synthesis of 24 peptides at a time on a 10 µmol resin scale using standard HCTU coupling chemistry. This is in contrast to standard SPPS, which is typically performed on a 0.1–0.2 mmol scale. These peptides were cleaved and reacted in crude form with 2 µM rFTase. Analysis of these peptides revealed a small number of hits. Of the 192 tested, only seven positive sequences (CITTL, CVHAL, CQTLI, CRFVT, CHSIA, CTSEI, CYLVK) were obtained. In our previous work, peptide hits were rescreened using an HPLC assay taking advantage of the fluorescent dansyl group on the N-terminus of the nonapeptides studied in the MALDI libraries to quantify the starting peptide; while the products could also be detected, their fluorescence varied substantially (compared to the starting peptide), making quantification problematic. With the peptides obtained in the current study from the human genome, this was even more problematic due to variations in the solubility of the peptides and their prenylated products (Figure S22). As an alternative, a continuous spectrofluorimetric assay was used that monitors the change in the Dansyl group fluorescence as the peptide is farnesylated [41]. For that assay, a larger fluorescence change is typically observed when the fluorophore is positioned closer to the site of prenylation and, hence, peptides bearing a simple Dansyl-Gly moiety upstream of the pentapeptide were prepared and studied. Unfortunately, those peptides react much more slowly, in part due to the absence of the upstream Arg residue present in the nonapeptides making it difficult to obtain kinetic constants (Figure S23). Upstream sequences containing cationic residues are known to enhance the affinity of substrate peptides for FTase, and it is possible that they may play a more important role in the recognition of extended pentapeptide sequences [42].

### 2.4. Validation of CaaaX Sequences Utilizing a Yeast Reporter Assay

**Figure 3:**
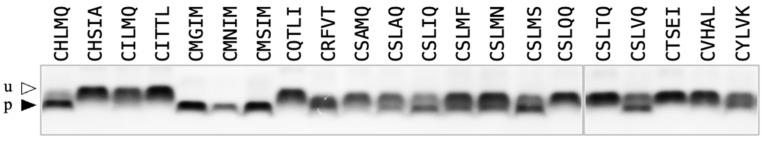
Mobility shift analysis of Ydj1p-CaaaX variants identified from peptide libraries. Whole-cell lysates prepared from yeast expressing the indicated Ydj1p-CaaaX variant were evaluated by SDS-PAGE and anti-Ydj1p immunoblot. The indicated Ydj1p variants were expressed in the humanized FTase yeast strain yWS3186 (*ydj1::NAT^R^ ram1::P_PGK_-FNTB his3∆1::HIS3-P_PGK_-FNTA*), which lacks naturally encoded Ydj1p and yFTase activity. Farnesylated Ydj1p exhibits a smaller apparent molecular mass relative to unmodified Ydj1p. Farnesylation profiles for the indicated Ydj1p-CaaaX variants were determined across multiple biological and technical replicates, from which the percent of farnesylated species relative to the total signal for a sample was determined (see Figure S24 and Table S2). Plasmids used in the analysis shown here are listed in Table S3.

To validate the prenylation of the extended CaaaX-box sequences discovered in the library experiments described above and circumvent the problems previously reported with the in vitro assays, we next evaluated them in a yeast reporter protein assay designed to measure their farnesylation levels in vivo. This assay also has the advantage that it allows the sequences to be studied in a more biologically relevant context. That assay involved the replacement of the endogenous tetrapeptide CaaX-box sequence found at the C-terminus of the protein Ydj1p, CASQ, with various pentapeptide CaaaX-box sequences. A total of 21 of the extended CaaaX-box hits from above were chosen for study, including positives obtained from libraries based on CMIIM and CSLMQ and sequences derived from the human genome analysis. The assay is based on the fact that proteins can exhibit a change in mobility in their migration in SDS-PAGE analysis upon farnesylation. Western blotting analysis using anti-Ydj1p antibodies and subsequent quantitation allows the calculation of the % gel-shifted protein, which is equal to the level of farnesylation. This assay was employed in our earlier study [37]. However, an important difference here was the use of a humanized yeast strain where the endogenous yFTase was replaced with the corresponding human enzyme [43]. This was carried out to make this analysis more relevant to prenylation in the human proteome. Representative data from that analysis are shown in Figure 3 and Figure S24, and the gel shift data are summarized in Table S2.

Analysis of the Ydj1-CaaaX variants revealed several important results. In most cases (17/21), some farnesylation (within the 95% confidence interval, see Table S2) was observed. However, accurate measurements of low levels of farnesylation using this assay are challenging given the large amount of the closely migrating unfarnesylated species. Hence, farnesylation levels below 20% should not be considered to be unambiguously positive. In general, a higher success rate was observed in analyzing the prenylation of sequences obtained from library screening. All three sequences derived from the CMa_1_IM parental sequence (CMGIM, CMNIM, and CMSIM) were found to be completely prenylated in vivo in the context of human FTase (hFTase). Most of the sequences derived from the CSLMQ parent sequence (CHLMQ, CSLIQ, CSLVQ, CSLAQ, CSLMS, CSLMF, and CSLMN) were modified to some extent. Interestingly, the sequence CHLMQ was not a hit in the library screens but was almost completely modified by hFTase. Because closely related CSLTQ and CSLQQ sequences were unmodified in vivo, this suggests that small hydrophobic amino acids (e.g., M, I, V, and A) are preferred at the a_2_ position by hFTase.

None of the hits selected from the human genome sequences were highly prenylated in the in vivo test. Since the amount of enzyme used in the library screening was almost certainly higher than levels present within yeast, the inability to observe prenylation of some of the MALDI-derived hits may simply reflect the fact that some of the hits are poor substrates. In some respects, this is not particularly surprising since those sequences derived from the libraries were based on parental sequences that were previously confirmed as substrates. In contrast, selections from the human genome represent new, unexplored sequence space. Nevertheless, these experiments provide an ever-increasing list of extended CaaaX-boxes that can be prenylated, thereby increasing the likelihood that proteins bearing these C-terminal extended CaaaX-box sequences may be prenylated in nature.

### 2.5. Crystal Structure of CMIIM Bound to CnFTase

**Figure 4:**
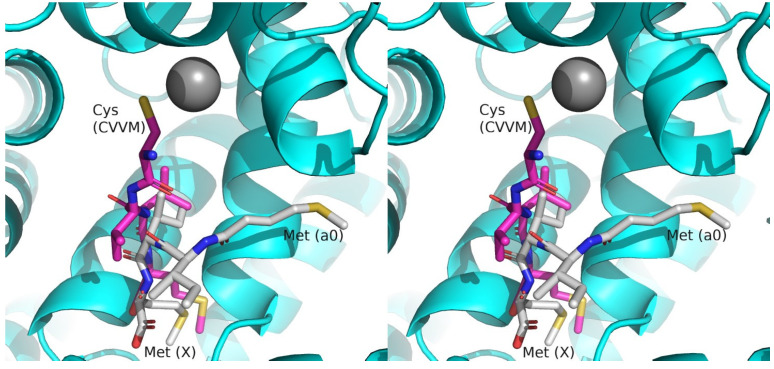
Stereo image of superposition of previously solved structure of the peptide CVVM (pink carbons) bound to CnFTase (cyan, pdb id 3q75) aligned with the newly reported structure of CMIIM (white carbons) bound to CnFTase (omitted for clarity, pdb id 8t70). Peptide atoms: N (blue); O (red); S (yellow); Zn (grey). The X positions in the Ca_0_a_1_a_2_X box are labeled. This image is provided in cross-eyed stereo. A parallel stereo image is shown in Figure S25.

**Figure 5:**
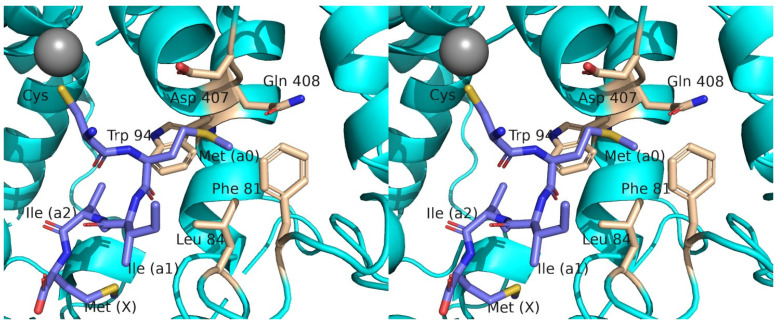
Stereo image of modeling of X-ray structure of CMIIM, modeling the previously undefined Cys residue to chelate Zn. The residues surrounding the a_0_ Met are shown in tan sticks and labeled. Peptide atoms: N (blue); O (red); S (yellow); Zn (grey). The Cys, a_0_, a_1_, a_2_, and X positions in the Ca_0_a_1_a_2_X box are labeled. This image is provided in cross-eyed stereo. A parallel stereo image is shown in Figure S27.

To improve understanding of peptapeptide recognition by FTase, an X-ray crystal structure of the peptide TKCMIIM bound to FTase from *Cryptoccoccus neoformins* (CnFTase) was obtained. For this, crystals of CnFTase were grown in the presence of TKCMIIM and the FPP analog FPT-II, and the structure of the ternary complex was obtained at 1.9 Å resolution (Table S4). The structure of the enzyme in this study is essentially identical to the protein in the CnFTase•CVVM•FPT-II ternary structure (Figure 4, Figure S25 and Figure S26) [44]. The FPT-II analog in these two structures is essentially superimposable as well. For the peptide ligands CMIIM and CVVM, the Met residues in the X-position align well with a rms deviation of 0.76 Å (overall heavy atoms). The Ile in the a_2_ position also aligns well with the Val with a rms deviation of 0.61 Å. At the a_1_ position, the structures diverge with a rms deviation of 1.66 Å, mainly driven by the displacement of the Cα (1.7 Å) and N (2.70 Å) at that position. For the CVVM structure, the thiol group from the Cys residue is coordinated to the active site Zn(II) atom in order to activate it for reaction with FPP. This Zn-S coordination has been observed in all FTase structures containing bound peptide substrates and is presumed to be a catalytically competent intermediate in the enzymatic reaction cycle [26,45,46]. In the CMIIM structure, the Met residue in the a_0_ position (corresponding to the Cys in CVVM) is oriented with the side chain directed into the ”exit groove” where the farnesyl group binds after it is translocated from the initial isoprenoid site following C-S bond formation and subsequent FPP binding for the next catalytic cycle (Figure 4, Figure S25 and Figure S26) [47]. The Met residue is in close proximity to several residues, including Phe81, Leu84, Trp94, Asp407, and Gln408, and interactions with those residues may confer specificity for what amino acids can be accommodated at the a_0_ position (Figure 5 and Figure S27). Finally, the Cys residue (in CMIIM) is not ligated to the Zn(II) ion but rather is disordered with what appears to be a thiophenol molecule (an impurity derived from peptide synthesis or crystallization reagents) bound to the open coordination site of Zn (II) instead.

While the solved X-ray structure complex does not completely show the catalytically competent species with the cysteinyl thiol bound as the Zn-thiolate, it provided the structural basis to further model the CMIIM pentapeptide complex bound to CnFTase. (Figure 5 and Figure S27).

### 2.6. Modeling and Molecular Dynamics Simulation

**Figure 6:**
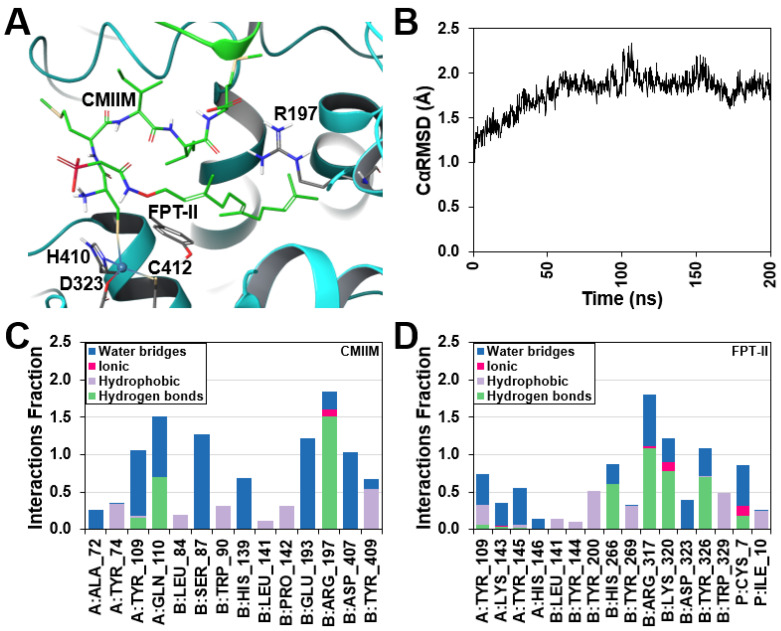
(**A**) Active site of CnFtase with bound CMIIM and FPT-II. (**B**) C_α_RMSD plot of CnFTase over the course of a 200 ns MD simulation. Interaction Fraction Plot for CMIIM (**C**) and FPT-II (**D**) within the CnFTase active site. The “A” and “B” prefixes in panels (**C**,**D**) refer to the alpha and beta subunits of CnFTase, respectively.

To better understand the molecular recognition process involved, a 200 ns Molecular Dynamics (MD) simulation of the modeled CnFTase (supplemental structural model file) in complex with CMIIM and FPT-II was carried out (Figure 6, Figure S28 and Figure S29). Given the intense interest in the development of protein prenyltransferase inhibitors [48], MD simulations [49,50] have provided valuable insights into these enzymes that are complementary to those obtained from the large number (over 100) of prenyltransferase X-ray crystal structures reported in the protein data bank (PDB). Here, an average Cα-RMSD of 1.79 Å with a maximum of 2.3 Å was observed throughout the course of the simulation with both CMIIM and FP remaining bound within the CnFTase site and the cysteine thiolate chelated to the catalytic Zn(II) ion (supplemental movie file). The interaction fractions highlighting the nature of protein–ligand interactions maintained over the course of the MD simulation are shown in Figure 6C,D. Given the hydrophobic nature of FPT-II and CMIIM, hydrophobic interactions play a critical role in their binding to CnFTase. Indirect water bridges between the ligands and the hydrophilic residues within the active site were also observed. The N-terminal amino group of CMIIM interacts directly with the terminal phosphate group of FPT-II. Most importantly, however, the simulation showed minimal movement of the C-terminal tripeptide relative to the crystallographic starting point with the C-terminal carboxylate group of CMIIM forming a direct salt bridge with the R197 side chain. Together, this suggests the combination of the cysteine chelation to the Zn(II) ion and the hydrophobic composition of the peptide and the C-terminal carboxylate group are required for the molecular recognition of the tetrapeptide and the extended pentapeptide CaaaX-box sequences.

### 2.7. Bioinformatic Analysis of Extended CaaaX-Box Recognition by FTase

**Figure 7:**
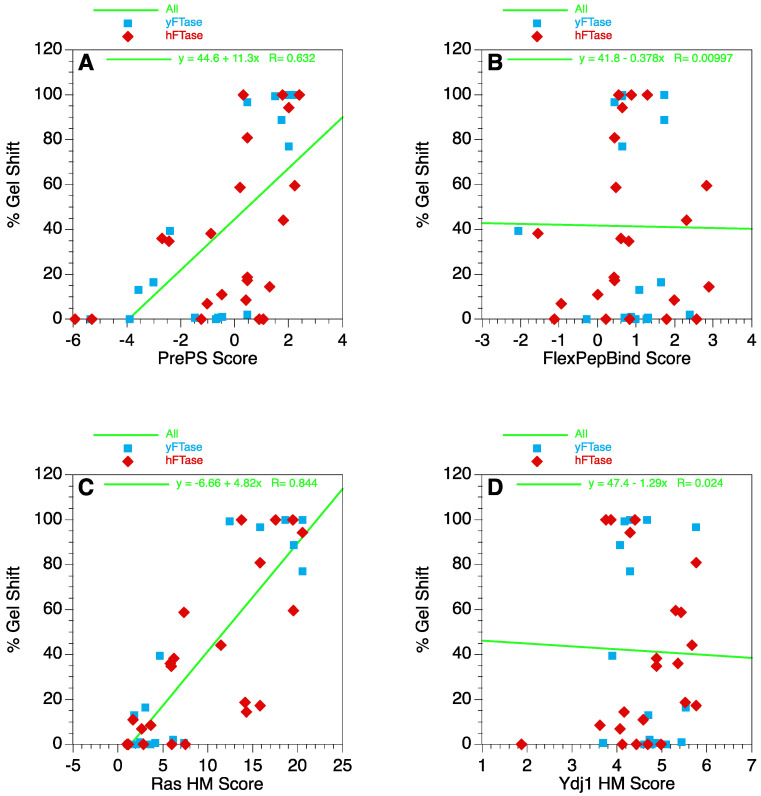
Comparison of different bioinformatic algorithms evaluated to correlate CaaX-box and extended CaaaX-box reactivity. (**A**) Analysis using PrePS. (**B**) Analysis using FlexPepBind. (**C**) Analysis using Ras HM. (**D**) Analysis using Ydj1 HM. Data points shown in red (hFTase) are from this study using Ydj1-CaaaX mutants evaluated in yeast expressing hFTase. Data points shown in blue (yFTase) are from a previous study using Ydj1-CaaaX mutants evaluated in yeast expressing endogenous yFTase [37]. The green line is the linear regression fit using all data (red and blue points).

The structural data reported above suggest that key interactions occurring between the C-terminal tripeptide and the enzyme in the context of canonical tetrapeptide substrates also occur in the recognition of extended pentapeptide sequences. If that is true, there should be a correlation between previously reported data regarding sequence selectivity for tetrapeptide CaaX-boxes and the data described here for the corresponding pentapeptides. Accordingly, several types of data were employed to probe this question. To accomplish that, the pentapeptide sequences were evaluated without the residue at the a_0_ position to generate a corresponding series of tetrapeptide sequences bearing the desired C-terminal tripeptides. Thus, for example, CMIIM was evaluated as CIIM where the underlined residue was ignored. First, PrePS, a web-based tool (https://mendel.imp.ac.at/PrePS/, accessed 1 June 2022) was used to calculate scores (see Table S5 for the resulting tetrapeptide sequences) [51]. That algorithm is based on a scoring system developed from a list of known prenylated proteins. PrePS scores were plotted versus the % gel shift data that reflect the extent of protein prenylation (Figure 3, Tables S2 and S5). That gel shift data included measurements for the 21 sequences reported here along with data from 18 sequences described in our previous work where the in vivo screening was performed using endogenous yFTase (39 sequences total). The analysis revealed a positive correlation of moderate significance (Figure 7A, r = 0.63). Next, a similar analysis was performed using the FlexPepBind algorithm developed by Schueler-Furman and coworkers for protein prenylation (Table S5) [52]. That method is based on FlexPepDock incorporated within the Rosetta modeling suite that was augmented using structural constraints obtained from crystallographic data from FTase–peptide complexes. A plot of FlexPepBind scores versus the % gel shift data noted above showed no correlation (Figure 7B, r = 0.010). A total of 2 other methods, based on a comprehensive analysis of yFTase specificity across all 8000 CaaX sequences, were also examined (Table S5); those methods are derived from a high-throughput analysis of farnesylated protein sequences obtained from a yeast thermotolerance screen where farnesylation of a heat shock protein is required for growth at an elevated temperature [23]. Of those two, the Ras HM (Heat Map) gave a strong correlation (Figure 7C, r = 0.84) while the Ydj1 HM gave no correlation (Figure 7D, r = 0.024). While the reasons for the poor correlation manifested by the FlexPepBind and Ydj1 HM algorithms are not yet understood, it is clear from these bioinformatic comparisons that there is a robust correlation between tetrapeptide and pentapeptide reactivity using PrePS and Ras HM and that this correlation could be used for predictive purposes in future studies.

### 2.8. Retrospective Analysis of 192 Human Sequences Studied Here

The bioinformatic analysis described above provides a useful model that can be employed to retrospectively predict the potential reactivity of the 192 human sequences that were screened in this study (Table S1). The PrePS correlation analysis (Figure 7A) suggests that sequences manifesting PrePS scores greater than 0.48 should be more than 50% prenylated. Of the 192 sequences examined, 16 had scores greater than 0.48. However, that analysis does not take into account the identity of the residue at the a_0_ position. To date, across all sequences identified as being prenylated in the gel shift assay, only H, I, M, S, and Y have been found in the a_0_ position at least twice, with L and Q found once. Examining the list of the 16 sequences predicted to be prenylated based on their PrePS scores reveals only 2 sequences with H, I, L, M, Q, S, or Y at the a_0_ position. One out of those two (a 50% hit rate) was actually observed to be prenylated in the gel-shift assay (CHSIA). A similar analysis using the Ras HM algorithm (Figure 6C) indicated that a score of at least 11.6 was required for 50% prenylation. In this case, of the 11 sequences meeting that criterion, only 4 have H, I, L, M, Q, S, or Y at the a_0_ position. A total of 1 out of those 4 (a 25% hit rate) was determined to be prenylated in the gel-shift assay. That sequence (CHSIA) was also found to be the top-scoring sequence that was observed to be prenylated in the gel-shift assay using PrePS. Importantly, the hit rates of 50% and 25% obtained using these bioinformatic predictions are substantially better than the 2.1% (4/192) obtained using our original selection criteria. In general, these results suggest that PrePS and Ras HM-based scoring approaches should be useful tools for the analysis of all possible pentapeptide sequences to provide a tractable number of sequences that could be analyzed experimentally.

## 3. Discussion

This work describes the continued evaluation of pentapeptide CaaaX sequences using a previously reported workflow employing focused libraries based on CaaaX sequences of interest. Libraries containing 10 sequences, chosen to eliminate isobaric overlap, obtained by randomizing a synthetic peptide at a single position were enzymatically farnesylated and evaluated by MALDI/MS. Libraries based on CMIIM, a sequence previously discovered and analyzed with yFTase, were further investigated utilizing rFTase, a homolog more relevant to human health (and less flexible in terms of substrate specificity) to evaluate differences between the two. Libraries based on CSLMQ, a pentapeptide positioned on the C-terminus of a human protein, transcription elongation factor A protein 3, were also analyzed using both yFTase and rFTase. Although it is unknown whether this protein is prenylated in vivo, our previous work and additional results presented here suggest it may be possible. To expand the repertoire of possible pentapeptide prenylation substrates, parallel synthesis coupled with MALDI-MS analysis was also used to evaluate 192 CaaaX sequences present in the human genome.

In analyzing the results from the CMIIM libraries reacted with rFTase at 2 µM enzyme, only variants at the a_1_ position showed prenylated products. This is in contrast to our previous findings using yFTase where there were hits at all four variable positions with the most at the a_0_ position, likely due to the fact that it is furthest away from the C-terminal X residue which is involved in the critical binding interactions. Increasing the concentration to 10 µM resulted in additional reactivity. Interestingly, the a_0_ position was still the least permissive to change with no hits observed by MALDI/MS. The X position showed a wider variety of substitutions using rFTase compared with yFTase, although a much higher concentration of enzyme was required. Well-known sequences including Gln, Ser, Cys, Met, and Ala, and more unusual ones such as His, Asn, Phe, and Glu were observed in that case. These additional hits indicate rFTase may have more flexibility for these extended sequences at the X position.

When analyzing the results with the CSLMQ libraries, we again found numerous differences between yFTase and rFTase. To our surprise, the normally more flexible yFTase yielded fewer hits than rFTase, most notably in the X position [33]. The yFTase results showed that only Asn and Gln were tolerated in the X position, while rFTase gave a mixture of hits, similar to the results from the CMIIM libraries. Interestingly, both enzymes displayed very low tolerance for amino acid substitutions in the a_0_ position, again in stark contrast to how yFTase behaves with CMIIM libraries. This highlights the importance of studying enzymes from different organisms and the use of multiple libraries, as a high number of hits at the a_0_ position appears to be an aberration instead of the rule. The a_1_ position is the most variable across all libraries with both enzymes showing a wide range of hits in each experiment. The a_2_ position for CSLMQ libraries gave more varied results, yielding 12 hits with rFTase and 6 hits with yFTase.

Analysis of the human genome for sequences of the type Ca_0_a_1_a_2_X identified over 1000 possibly prenylatable proteins. We evaluated 192 unique Ca_0_a_1_a_2_X sequences by utilizing parallel synthesis and evaluating crude peptide material via MALDI-MS analysis. Of the 192, only CITTL, CVHAL, CQTLI, CRFVT, CHSIA, CTSEI, and CYLVK, displayed any reactivity. When those 7 sequences were screened in a humanized yeast strain, 4 of them were found to be prenylated, including CITTL (derived from voltage-dependent L-type calcium channel subunit alpha-1D), CQTLI (derived from CBY1-interacting BAR domain-containing protein 1), CHSIA (derived from CDAN1-interacting nuclease 1) and CYLVK (derived from coiled-coil domain-containing protein 144A). Given that less than 20% of the possible Ca_0_a_1_a_2_X sequences present in the human genome were screened in this study, these observations suggest that there could be considerably more putative farnesylated substrates in the human genome as well as in other organisms.

The acquisition of a high-resolution X-ray crystal structure provided important insight into the mode of binding for pentapeptide sequences to FTase. Based on the substantial overlap of the structures of a CaaX-box (CVVM) and extended CaaaX-box peptide substrate (CMIIM), it appears that the C-terminal tripeptides from each bind in a similar fashion. A 200 ns molecular dynamic simulation of the complex suggested that the common tripeptide conformation was maintained when the peptide-derived thiol was bound to the active site Zn(II), as required for catalysis. Bioinformatic analysis focused on the relationship between tetrapeptide and pentapeptide prenylation indicates that there is a strong correlation between the two, reinforcing the relevance of the structural model. Together, those data strongly suggest that CaaaX-box recognition can be largely predicted based on the identity of the C-terminal tripeptide although the identity of the residue at the a_0_ position still likely plays a key, although less defined, role. Overall, the robust workflow described here involving initial screening of synthetic peptides via MALDI-MS and confirmation in a cell-based system has led to the discovery of several possible prenylated proteins in the human proteome. Additionally, based on a structural model, bioinformatic analysis has yielded insights that should be useful for the identification of additional prenylated proteins in future studies.

## 4. Materials and Methods

### 4.1. Library Synthesis 

Peptide libraries were synthesized using Fmoc-based solid-phase peptide synthesis (SPPS) employing a Gyros Protein Technologies PS3^®^ peptide synthesizer (Uppsala, Sweden) using four equivalents of Fmoc-protected amino acids from Aldrich^®^ (St. Lous, MO, USA), Novabiochem^®^ (Burlington, MA, USA) and P3 Biosystems (Louisville, KY, USA), and Fmoc-AA-Wang resins from P3 Biosystems. HCTU was used as the coupling reagent (0.4 mM) and 0.8 M DIEA was used as the base. The synthesis of libraries containing 10 different amino acids per library was performed by varying the “X” position. Manual coupling of Dansyl-Gly (DsG) was performed using a two-fold molar excess and a reaction time of 4–6 h. Upon completion of the synthesis, peptides were cleaved from resin with Reagent K (82.5% trifluoroacetic acid (TFA), 5% thioanisole, 5% phenol, 2.5% 1,2-ethanedithiol, and 5% H_2_O, *v*/*v*) cleavage cocktail using 5 mL per 0.1 mmol of resin for 2 h. The resulting peptides were precipitated from the cleavage solution using 40 mL Et_2_O cooled in an isopropanol/dry ice bath for ~10 min, collected via centrifugation, and washed once with additional cold Et_2_O (40 mL) to remove residual cleavage reagents. The crude solid was then dissolved in 50:50 CH_3_CN/H_2_O containing 0.1% TFA and the peptide concentration was determined by measuring the absorptivity at 338 nm using the molar extinction coefficient of Dansyl-Gly (4300 cm^−1^ M^−1^). Peptide hits from MALDI screening were resynthesized using similar conditions. Peptide libraries, with the exception of CSLMX, were synthesized where the variable position X = C, F, G, I, K, M, N, S, V, and Y for Library 1 and X = A, D, E, H, L, P, Q, R, T, and W for Library 2. In the case of CSLMX libraries, X = C, D, F, G, I, K, M, S, V, Y for Library 1 and X = A, E, H, L, N, P, Q, R, T, and W for Library 2. A complete tabulation of all amino acids varied in each library as well as observed prenylated hits can be found in Table S6.

### 4.2. General Enzymatic Farnesylation of Peptides

Enzymatic farnesylation of peptide libraries was performed by incubating FTase from *S. cerevisiae* (yFTase) or *R. norvegicus* (rFTase) in a reaction buffer that contained peptide (20 μM total), FPP (40 μM), Tris-HCl pH 7.5 (50 mM), ZnCl_2_ (10 μM), MgCl_2_ (5 mM), and DTT (1 mM) in H_2_O [53,54]. Reactions were allowed to proceed at 37 °C for 5 h. Upon completion, the samples were desalted using a reverse-phase C18 environmental cartridge (Waters Corporation, WAT023635, 3 cm × 1 cm diameter) (Milford, MA, USA). Cartridges were primed using 3 mL of Buffer B (CH_3_CN with 0.1% TFA) followed by equilibration with 3 mL of Buffer A (H_2_O with 0.1% TFA). The sample was loaded, washed with 2 mL each of 100% Buffer A, 10% Buffer B in Buffer A, and 20% Buffer B in Buffer A, followed by elution with Buffer B (2 mL). Samples were either immediately spotted on a MALDI plate or stored at −80 °C for further use Control libraries were treated and analyzed under the same conditions but without the addition of FTase.

### 4.3. MALDI-TOF MS of Farnesylated Peptide Libraries

Samples purified as described above (0.5 μL) were co-spotted with an identical volume of 10 mg/mL α-cyano-4-hydroxycinnamic acid (CHCA) matrix dissolved in a 50:50 mixture of Buffer A and Buffer B (as defined above) on an TOF plate (AB Sciex 384 Opti). The typical spotting procedure involved spotting the matrix first, then immediately spotting the sample on top of the matrix, followed by rapid pipetting up and down to mix. In contrast to the HPLC experiments described below, enzymatic reaction mixtures were not centrifuged prior to subsequent co-crystallization with the matrix. Samples were then analyzed with a MALDI/TOF mass spectrometer using the reflector positive mode (AB-Sciex 5800 13) (Framingham, MA, USA). A laser intensity of ~4000–5000 was employed using a pulse rate of 400 Hz. The laser intensity was increased in increments of 200 if the signal was not readily observable. A total of 4000 laser shots were applied per spectrum to ensure that the entire spot surface was sampled. For analysis, peaks in the *m*/*z* region corresponding to the masses of prenylated products were initially filtered using a signal-to-noise ratio of 12 as a cutoff, and resulting peaks corresponding to the expected mass of prenylated and unprenylated peptides were labeled. Occasionally, peaks originating from incomplete peptide deprotection were observed in this region.

### 4.4. HPLC Based Enzymatic Farnesylation Assay

Enzymatic farnesylation reactions with purified peptides were performed by incubating FTase from *S. cerevisiae* (yFTase) or *R. norvegicus* (rFTase) in a reaction buffer that contained peptide (2.4 μM), 1 FPP (10 μM), Tris-HCl pH 7.5 (50 mM), ZnCl_2_ (10 μM), MgCl_2_ (5 mM), and DTT (1 mM) in H_2_O [53,54]. Prior to the enzymatic reaction, peptide stock solutions were incubated in 5 mM DTT for 30 min at rt to ensure complete thiol reduction. Reactions with yFTase were performed at rt for 30 min while, reactions with rFTase were carried out at 35 °C for 45 min. The reactions were stopped using flash freezing. Prior to analysis, the samples were thawed, and centrifuged to remove particulate matter, followed by injection (200 µL) into an Agilent (Santa Clara, CA, USA) 1100 HPLC instrument equipped with a fluorescence detector and a Phenomenex (Torrance, CA, USA) Luna 5-micron C18 100 Å pore size 250 × 4.60 mm, 5 µm analytical column equilibrated in 99% Buffer A/1% Buffer B (as defined above). A flow rate of 1 mL/min was used, and the gradient was either 1% to 100% Buffer B over 25 min with 5 min delay at the beginning of method (Figures S8–S10) or 1% to 100% Buffer B over 40 min with a 5 min delay at the beginning of the method (Figures S21 and S22). The fluorescence of the dansylated peptides was monitored with an excitation of 220 nm and an emission of 495 nm using a PMT gain of 12. Those detection conditions were based on previously reported efforts described in the literature using 220 nm excitation [55,56,57] as well as a study of dansylglycine fluorescence performed in different Buffer A/Buffer B compositions. Excitation and emission spectra for that study are provided in Figure S30. All reactions were run in triplicate. The extent of conversion was quantified by integrating the peak area from the starting material present in the HPLC fluorescence detector chromatogram. Control reactions containing all buffer components except enzyme were used to confirm that the observed decreases in starting peptide were enzyme dependent and not due to starting material loss during sample processing including filtration prior to HPLC.

### 4.5. Peptide Search of the Human Proteome

The UniProtKB was examined using the scanProsite tool of Expasy to identify known protein sequences that contain a potential pentapeptide CaaaX sequence (https://prosite.expasy.org/scanprosite/, accessed 1 June 2022). The search was restricted to C-terminal sequences Ca_0_a_1_a_2_X, where all 20 canonical amino acids were allowed in the a_0_, a_1_, a_2_, and X positions. Isoforms were included in the motif search. The results from that initial search were then filtered to limit the hits to sequences present in the human proteome (*H. sapiens*). Additional filtering was performed to remove sequences that contained multiple charged residues, multiple conformationally challenging residues (Gly and Pro), or one of each from the aforementioned categories.

### 4.6. Synthesis of Peptides Derived from the Human Genome

Peptides were synthesized using Fmoc-based solid-phase peptide synthesis (SPPS) using an Intavis MultiPep RS^®^ peptide synthesizer (Uppsala, Sweden) using four equivalents of Fmoc-protected amino acids from Aldrich^®^ (St. Lous, MO, USA), Novabiochem^®^ (Burlington, MA, USA) and P3 Biosystems (Louisville, KY, USA), and Fmoc-AA-Wang resins from P3 Biosystems. Coupling was performed with standard HCTU coupling procedures, (0.4 mM), and 0.8 M DIEA was used as the base, with Dansylglycine being allowed to react for an extended 4 h time. Upon completion of the synthesis, peptides were cleaved from resin with Reagent K (82.5% trifluoroacetic acid (TFA), 5% thioanisole, 5% phenol, 2.5% 1,2-ethanedithiol, and 5% H_2_O, *v*/*v*) cleavage cocktail using 5 mL per 0.1 mmol of resin for 2 h. The resulting peptides were precipitated from the cleavage solution using 40 mL Et_2_O, cooled in an isopropanol/dry ice bath for ~10 min, collected via centrifugation, and washed once with additional cold Et_2_O (40 mL) to remove residual cleavage reagents. The crude peptides were then dissolved in 50:50 CH_3_CN/H_2_O containing 0.1% TFA and evaluated by enzymatic reaction and MALDI analysis as described above.

### 4.7. Determination of Kinetic Parameters for Dansyl-GCSLMQ

Fluorescence of prenylation by 100/200 nM rFTase of 1.0, 2.0, 5.0, and 10.0 μM Dns-GCSLMQ, was monitored over time with 10 μM farnesyl pyrophosphate (FPP) and 5 mM MgCl_2_. In a black 96-well plate (Corning, NY, USA) peptide in 1X reaction buffer (50 mM HEPPSO-NaOH (pH 7.8) and 5 mM TCEP) was incubated for 20 min, in the dark. Enzyme and FPP were mixed in 1X reaction buffer (50 μL) before the addition in the black 96-well plate to initiate the reaction. Fluorescence was collected using a BioTek (Santa Clara, CA, USA) H1 Synergy Plate Reader (λex = 340 nm and λem = 520 nm). Initial velocities were obtained by fitting the data at the beginning of the reaction to a linear equation, giving rates in units of fluorescence per second (Fl/s). Steady-state kinetic parameters were obtained using an amplitude conversion to convert fluorescence to µM by dividing the total fluorescence reached upon completion (ΔF) by the dansylated peptide concentrations to obtain Fl/μM. Initial velocity data (F/s) were then divided by this number to obtain the velocity of the reaction (μM/s). Velocities calculated were plotted against peptide concentrations (μM) and fitted to a Michaelis–Menten curve. Kaleida Graph, version 4.5.4 (Synergy Software, Reading, PA, USA) was used for curve fitting.

### 4.8. Yeast Strains and Plasmids

Standard yeast genetic manipulations were used to construct the humanized FTase yeast strain yWS3186 (MAT**a** leu2∆0 met15∆0 ura3∆0 his3∆1::HIS3-P_PGK_-FNTA ram1::P_PGK_-FNTB ydj1::NAT^R^) [43]. The human FTase α and β subunits (FNTA and FNTB, respectively) were integrated into the genome of a ydj1∆ yeast strain (BY47441 background) behind the yeast phosphoglycerate kinase (PGK) promoter. P_PGK_-FNTA was integrated at the his3∆1 locus by homologous recombination using an HIS3-based integrative plasmid. P_PGK_-FNTB was integrated at the RAM1 locus, replacing the open reading frame, using a loop-in loop-out strategy. All gene replacements were verified by PCR. Plasmids encoding Ydj1-CaaaX variants were introduced into strains via a lithium acetate-based transformation procedure [58]. Plasmid-transformed yeast were propagated at rt in selective media (i.e., SC-Uracil). Plasmids were created by PCR-directed recombination-mediated cloning [59,60]. In brief, yeast cells were co-transformed with NheI-digested pWS1132 (CEN URA3 YDJ1-SASQ) and a PCR product encoding the desired CaaaX sequence flanked by 5′ and 3′ sequences that were identical to regions of pWS1132 to facilitate homologous recombination. Candidate plasmids were recovered from yeast, amplified in *E. coli*, and DNA sequencing was used to confirm the YDJ1-CaaaX open reading frame.

### 4.9. Mobility Shift Analysis of Ydj1 Farnesylation

Whole-cell lysates were prepared from yeast cultured to approximately 1 A_600_ in SC-Uracil liquid media at rt as previously described [37,59]. Protein samples were analyzed by SDS-PAGE (9.5%) followed by Western blotting with rabbit anti-Ydj1 primary antibody (courtesy of Dr. Avrom Caplan) and HRP-conjugated goat anti-rabbit secondary antibody (Kindle Biosciences, Greenwich, CT, USA). Immune complexes were detected using WesternBright TM ECL-spray (Advansta, San Jose, CA, USA) and a KwikQuant Imager (Kindle Biosciences, Greenwich, CT, USA) at multiple exposure times. Levels of farnesylation were quantified for multiple replicates using ImageJ software, version 1.54i.

### 4.10. Crystallization, Data Collection, and Structure Determination

Protein expression and purification. The CnFTase protein was expressed and purified as described previously [61]. The purified protein was concentrated to approximately 10 mg/mL using a centrifugal concentrator (50 kDa cutoff), exchanged into long-term storage buffer (20 mM HEPES, pH 7.5, 5 μM ZnCl_2_, 5 mM DTT followed by flash freezing in liquid nitrogen, and storage at −80 °C.

Crystallization. Crystals of the ternary complex of CnFTase, FPP analog (FPT-II), and peptides were determined as described previously [44,61]. Here, CnFTase (10 mg/mL in storage buffer) was mixed with tris [2-carboxyethyl] phosphine (TCEP) pH 7.5 to a final concentration of 5 mM TCEP. Protein was pre-incubated with a 1.5-fold molar excess of the FPP analog FPT-II (Sigma) for 30 min on ice. Peptide TKCMIIM (Genscript, Piscataway, NJ, USA) was dissolved in DMSO and added to protein FPT-II complex in 3-fold molar excess. Crystals were grown at 17 °C by hanging-drop vapor diffusion (1 µL protein drop, 0.5 µL reservoir of 100 mM CAPSO pH 9.5, 50–75 mM Li_2_SO_4_, 200 mM NaCl, 16–21% PEG4K). Crystal seeds prepared from CnFTase-FPTII crystals were added to drops to accelerate the crystal growth. The resulting crystals were transferred stepwise into a cryoprotection solution (well solution plus ~30% ethylene glycol) and flash-frozen in liquid nitrogen.

Data collection and structure determination. X-ray diffraction data were collected at SER-CAT Beamline 22-ID at the Advanced Photon Source, Argonne National Laboratory. The crystals belonged to the space group P43212 with the unit cell dimensions 141 Å × 141 Å × 130 Å with 1 CnFTase heterodimer in the asymmetric unit. The ternary complex crystals diffracted to approximately 1.9 Å resolution. Structures were solved as described previously [26,44,61]. The peptide was fit into different electron density maps. The first four C-terminal residues were clearly defined by the density with the remaining residues being only partially disordered. Additional “mystery” density consistent with the small molecule thiophenol was observed to coordinate the zinc ion.

### 4.11. Modeling and Molecular Dynamics Simulation

The complex including the CMIIM pentapeptide and FPT-II bound to CnFTase was modeled using Schrödinger (New York, NY, USA) Modeling Suite Package based on the current solved X-ray structure, with the cysteine coordinates derived from TKCVVM-CnFTase (PDB: 3Q75) [61,62]. Missing hydrogen atoms were added according to the predicted pKa of ionizable amino acid residues at pH 7.0 using PropKa, followed by energy minimization using the OPLS4 force field [63,64]. The structure was then solvated using TIP3P explicit water in an orthorhombic box with 12 Å buffer region and electroneutralized with 0.15 M Na^+^ and Cl^−^ counterions [65]. Molecular dynamics (MD) simulation was carried out using Desmond with default relaxation protocol followed by 200 ns production simulation under NPT ensemble conditions at 300 K and 1 atm [66]. Analysis of the simulation was carried out using Schrodinger’s Simulation Interactions Diagram (SID) tool.

### 4.12. Bioinformatic Analysis

For bioinformatic analysis, all pentapeptide sequences were evaluated without the residue at the a_0_ position. As an example, CMIIM was evaluated as CIIM where the underlined residue was ignored. For PrePS, the 11-residue sequence GKKKKKKSKTK, derived from the C-terminus of K-Ras, was appended to the N-terminus of the CaaX-box to yield a 15-residue sequence. This was necessary since PrePS cannot be used on simple tetrapeptides. For FlexPepBind, Ras HM, and Ydj1 HM, the tetrapeptide sequence was sufficient for conducting analyses.

## Figures and Tables

**Table 1:** Normalization of hits from DsGRAGCMa_1_IM Library 1 shared between rFTase and yFTase.

Amino Acid	rFTase	yFTase	rFTase:yFTase
Gly	0.72	0.29	2.5
Ser	0.66	0.46	1.4
Asn	0.55	0.38	1.4
Gln	0.49	0.48	1.0
His	0.31	0.63	0.49

**Table 2:** Summary of peptides observed in CMIIM MALDI/MS libraries using rFTase at 10 µM.

Library Sequence	Observed Amino Acid Hits
Ca_0_IIM	n.o. ^a^
CMa_1_IM ^b^	A, C, E, G, H, M, N, Q, R, S, T, Y
CMIa_2_M	A, C, D, E, F, H, L, M, N, Q, S, T, V, Y
CMIIX	A, C, E, F, H, M, N, Q, S, T

^a^ No hits were observed at this position (n.o., none observed); ^b^ these libraries showed conversion at 2 µM, while all others had no prenylation at this concentration. Reactions were run at 35 °C for 8 h. Peptides were synthesized and tested with a DsGRAG sequence upstream of the CaaaX box.

**Table 3:** Summary of peptides observed in CSLMQ MALDI/MS libraries using yFTase at 1 µM.

Library Sequence ^a^	Observed Amino Acid Hits
Ca_0_LMQ	C, S, V
CSa_1_MQ	A, C, D, E, F, G, L, M, N, P, Q, R, S, Y
CSLa_2_Q	A, L, M, Q, T, V
CSLMX	N, Q

^a^ Reactions were run at 35 °C for 8 h. Peptides were synthesized and tested with a DsGRAG sequence upstream of the CaaaX box.

**Table 4:** Summary of peptides observed in CSLMQ MALDI/MS libraries using rFTase at 3 µM.

Library Sequence ^a^	Observed Amino Acid Hits
Ca_0_LMQ	I
CSa_1_MQ	A, C, D, G, L, M, N, P, Q, R, S, T, V, Y
CSLa_2_Q	A, C, E, F, G, I, L, M, S, V, W, Y
CSLMX	A, C, D, E, F, I, M, N, Q, S, T, Y

^a^ Reactions were run at 35 °C for 8 h. Peptides were synthesized and tested with a DsGRAG sequence upstream of the CaaaX box.

## Data Availability

Data are contained within the article and Supplementary Materials.

## Supplementary Materials

The following supporting information can be downloaded at: https://www.mdpi.com/article/10.3390/ijms25105324/s1.
