# Supplementary material for: Library Screening, In Vivo Confirmation, and Structural and Bioinformatic Analysis of Pentapeptide Sequences as Substrates for Protein Farnesyltransferase
Source: Int J Mol Sci. 2024 May 13;25(10):5324. doi: 10.3390/ijms25105324 (PMC11121372; doi:10.3390/ijms25105324)
Supplement: Supplementary file 1 [file ijms-25-05324-s001.zip › ScheyIJMS_05064_SI.pdf]

Library Screening, In Vivo Confirmation, and Structural and Bioinformatic Analysis of Pentapeptide Sequences as Substrates for Protein Farnesyltransferase

Garrett L. Schey[1], Emily R. Hildebrandt[2], You Wang[3], Safwan Diwan[4], Holly A. Passetti[4], Gavin W. Potts[4], Andrea M. Sprague-Getsy,[5] Ethan R. Leoni[2], Taylor S. Kuebler[6,7], Yuk Y. Sham[6,7], James L. Hougland[5,8,9], Lorena S. Beese[3], Walter K. Schmidt[2], Mark D. Distefano[1,4*]

[1]Department of Medicinal Chemistry, University of Minnesota, Minneapolis, Minnesota 55455, USA; schey013@umn.edu, diste001@umn.edu

[2]Department of Biochemistry and Molecular Biology, University of Georgia, Athens, Georgia 30602, USA; erh@uga.edu, erl99598@uga.edu, wschmidt@uga.edu

[3]Department of Biochemistry, Duke University School of Medicine, Durham, North Carolina 27710, USA; you.wang@duke.edu, lorena.beese@duke.edu

[4]Department of Chemistry, University of Minnesota, Minneapolis, Minnesota 55455, USA; diwan005@umn.edu, hap5160@psu.edu, gpotts80813@gmail.com, diste001@umn.edu

[5]Department of Chemistry, Syracuse University, Syracuse, New York 13244 USA; anspragu@syr.edu, hougland@syr.edu

[6]Department of Integrative Biology and Physiology, Minneapolis, Minnesota 55455, USA; kuebl010@umn.edu, shamx002@umn.edu

[7]Bioinformatics and Computational Biology Graduate Program, Minneapolis, University of Minnesota, 55455, USA; kuebl010@umn.edu, shamx002@umn.edu

[8]Department of Biology, Syracuse University, Syracuse, New York 13244 USA; hougland@syr.edu

[9]BioInspired Syracuse, Syracuse University, Syracuse, New York 13244 USA; hougland@syr.edu

*****Correspondence: diste001@umn.edu

Table of Contents

Figures

Figure S1. Analysis of CMa_1IM library 2 screened with rFTase. 4

Figure S2. Analysis of Ca_0IIM library 1 screened with rFTase. 5

Figure S3. Analysis of the Ca_0IIM library 2 screened with rFTase. 6

Figure S4. Analysis of $CMIa_2M$ library 1 screened with rFTase. 7

Figure S5. Analysis of $CMIa_2M$ library 2 screened with rFTase. 8

Figure S6. Analysis of CMIIX library 1 screened with rFTase. 9

Figure S7. Analysis of CMIIX library 2 screened with rFTase. 10

Figure S8. HPLC assay of CMGIM. 11

Figure S9. HPLC assay of CMNIM...11

Figure S10. HPLC assay of CMSIM. 12

Figure S11. Analysis of CSLMQ a_0 and a_1 libraries screened with yFTase. 12-13

Figure S12. Analysis of CSLMQ a_2 and X libraries screened with yFTase. 14-15

Figure S13. Analysis of Ca_0LMQ library 1 screened with rFTase. 16

Figure S14. Analysis of Ca_0LMQ library 2 screened with rFTase. 17

Figure S15. Analysis of CSa_1MQ library 1 screened with rFTase. 18

Figure S16. Analysis of CSa_1MQ library 2 screened with rFTase. 19

Figure S17. Analysis of $CSLa_2Q$ library 1 screened with rFTase. 20

Figure S18. Analysis of $CSLa_2Q$ library 2 screened with rFTase. 21

Figure S19. Analysis of CSLMX library 1 screened with rFTase................................ 22

Figure S20. Analysis of CSLMX library 2 screened with rFTase................................ 23

Figure S21. HPLC assay of CSLMQ. 24

Figure S22. HPLC assay of CMSIM. 24

Figure S23. Kinetic analysis of CSLMQ 25

Figure S24. Western blot analysis of Ydj1-CaaaX samples 26

Figure S25. Alternative stereo image version of Figure 4. 27

Figure S26. Stereoview of superposition of TKCVVM and CMIIM bound to CnFTse. . 27

Figure S27. Alternative stereo image version of Figure 5. 28

Figure S28. Stereoview of superposition crystal structure of CMIIM before and after

modeling of the Cys to coordinate the active site Zn. 28
Figure S29. MD simulation of CMIIM bound to CnFTase.. 29
Figure S30. Excitation spectra of Dansyl Glycine. 30

Tables

Table S1. List of 192 CaaaX sequences synthesized from the human genome and their calculated PrePS and Ras HM scores based on their three C-terminal residues. .... *Separate File*

Table S2. Summary of gel-shift data obtained from Western blotting of extracts obtained by expression of CaaaX-box sequences fused to the C-terminus of Ydj1. 24

Table S3. Plasmids used in these studies. 25

Table S4. Summary of data collection and refinement information for the crystal structure of TKCMIIM and FTPII bound to CnFTase. 26

Table S5. List and bioinformatic analysis of all CaaaX-box sequences selected for further study after MALDI screening. .... *Separate File*

Table S6. List of amino acids in variable positions in each library as well as observed prenylated hits….. .... *Separate File*

Supplemental Movie File.. .... *Separate File*

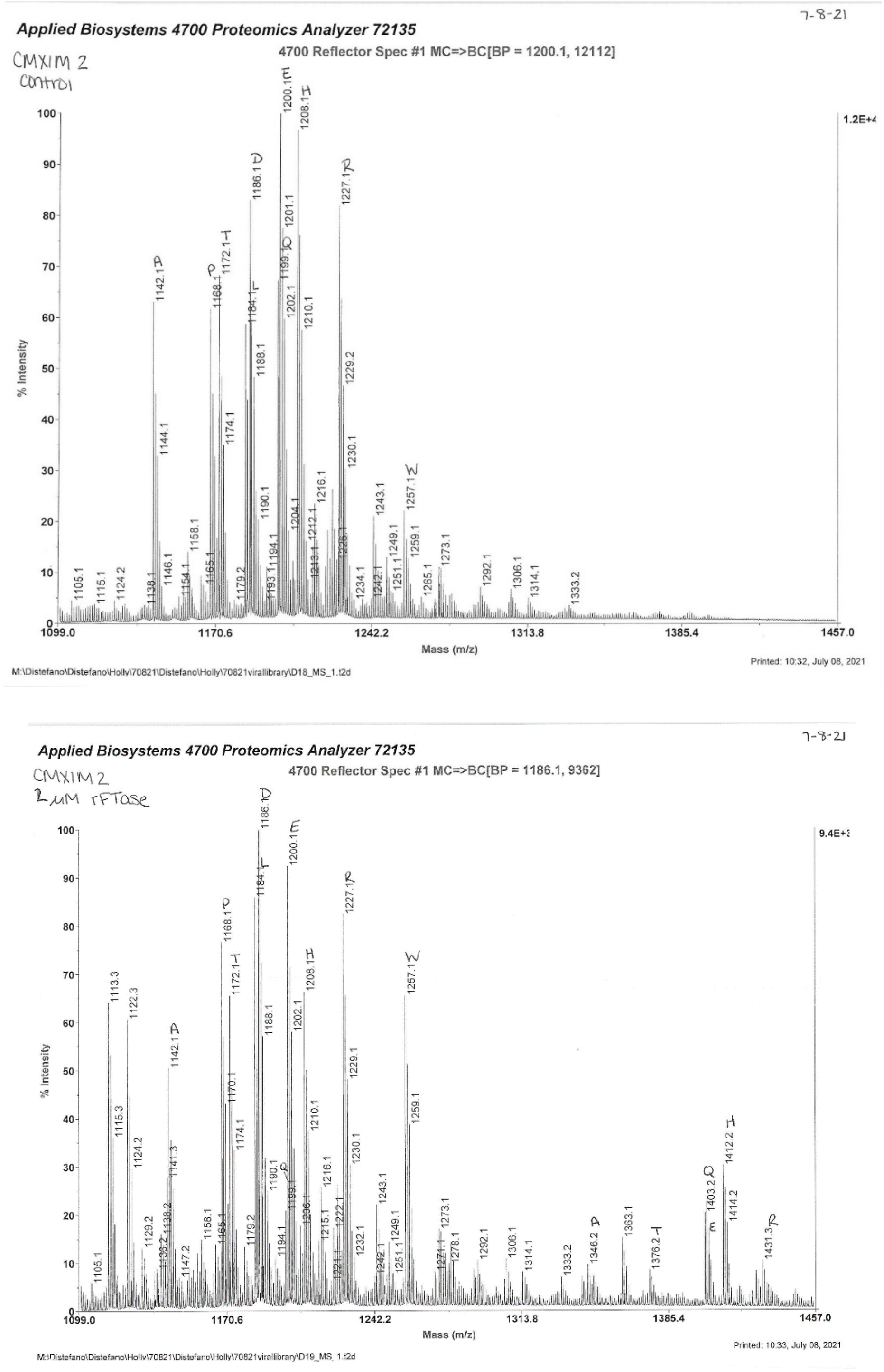

Figure S1. Analysis of CMa$_1$IM library 2 screened with rFTase before (above) and post reaction (below). The reaction was performed using 2 μM enzyme and analyzed by MALDI-MS.

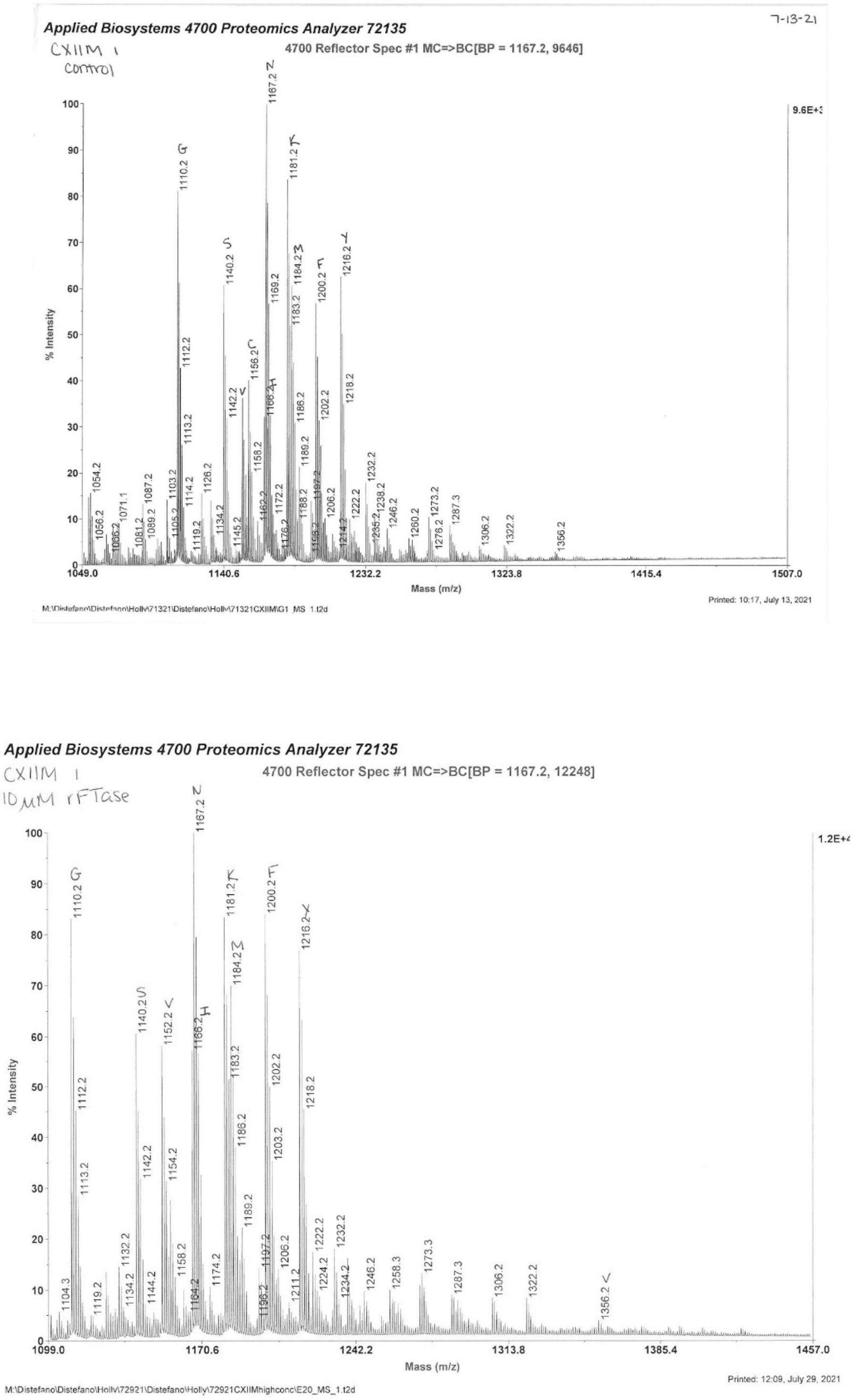

Figure S2. Analysis of Ca_0IIM library 1 screened with rFTase before (above) and post reaction (below). The reaction was performed using 10 µM enzyme and analyzed by MALDI-MS.

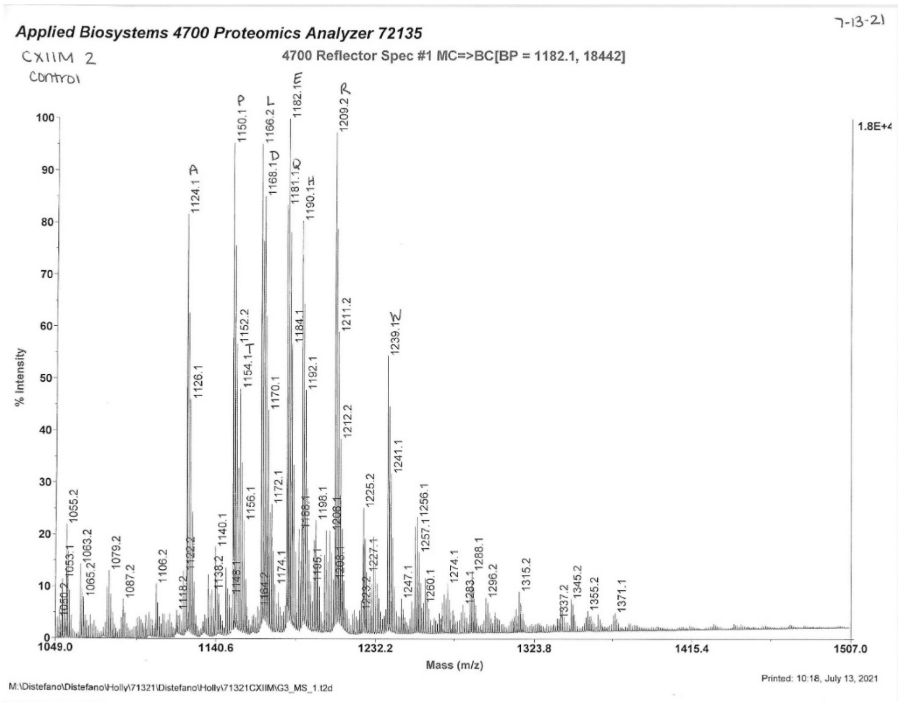

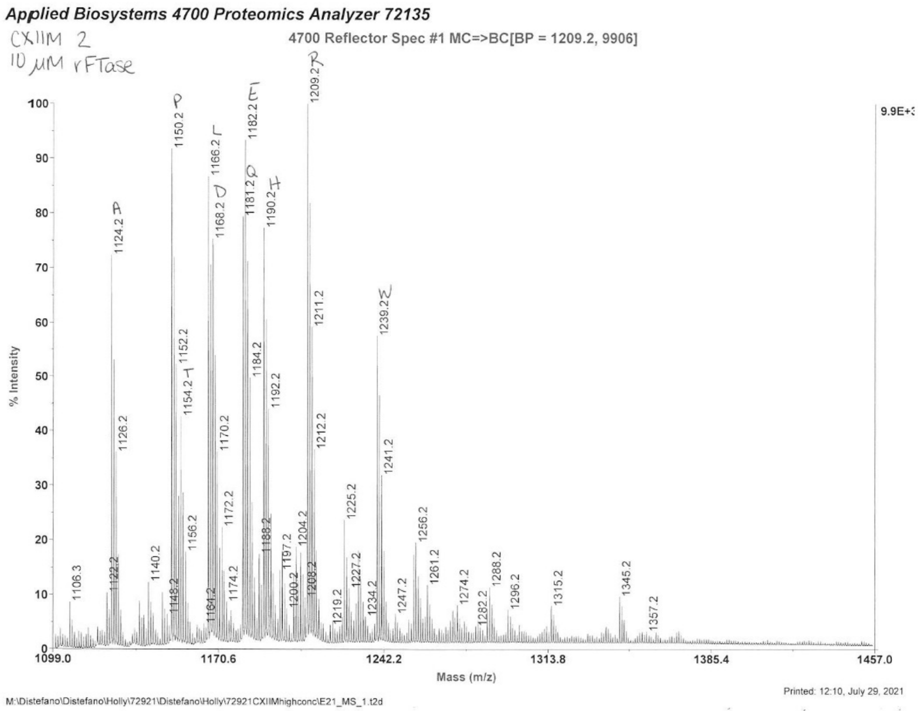

Figure S3. Analysis of Ca_0IIM library 2 screened with rFTase before (above) and post reaction (below). The reaction was performed using 10 μM enzyme and analyzed by MALDI-MS.

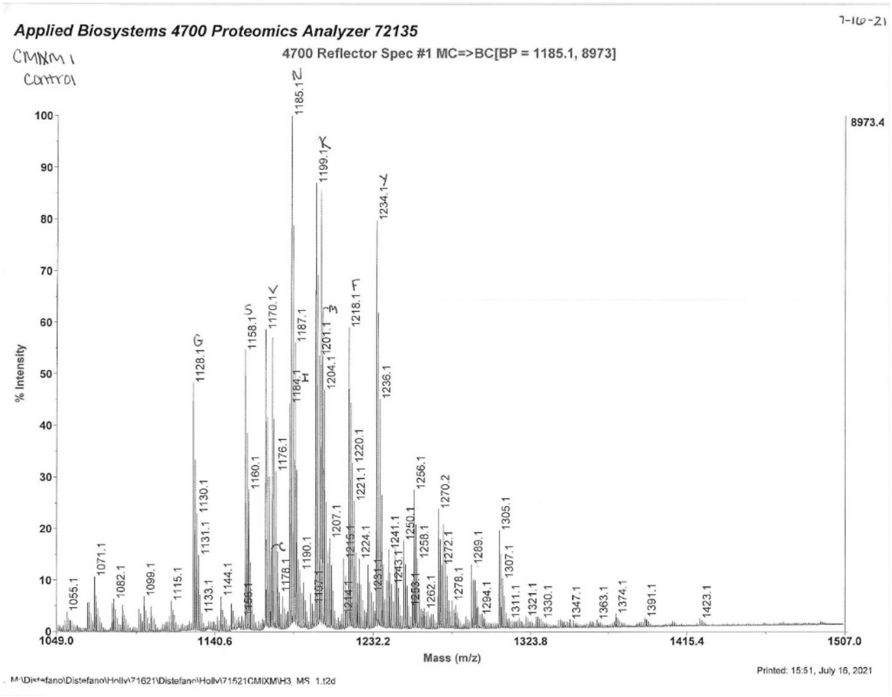

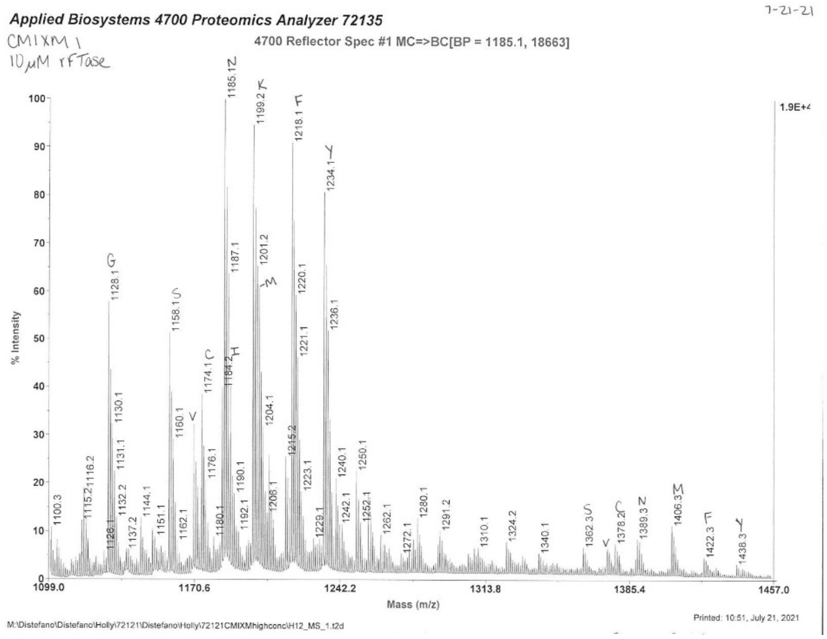

Figure S4. Analysis of CMIa$_2$M library 1 screened with rFTase before (above) and post reaction (below). The reaction was performed using 10 µM enzyme and analyzed by MALDI-MS.

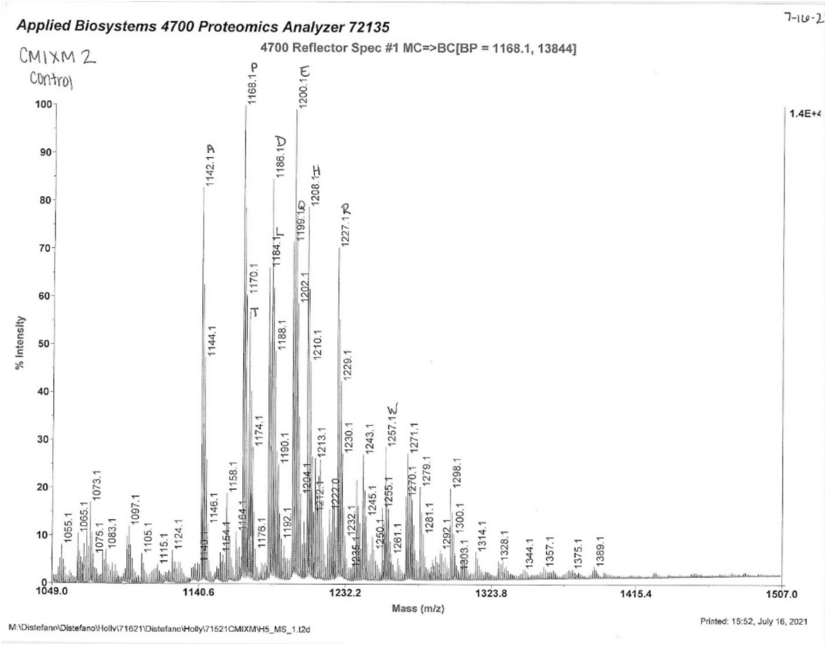

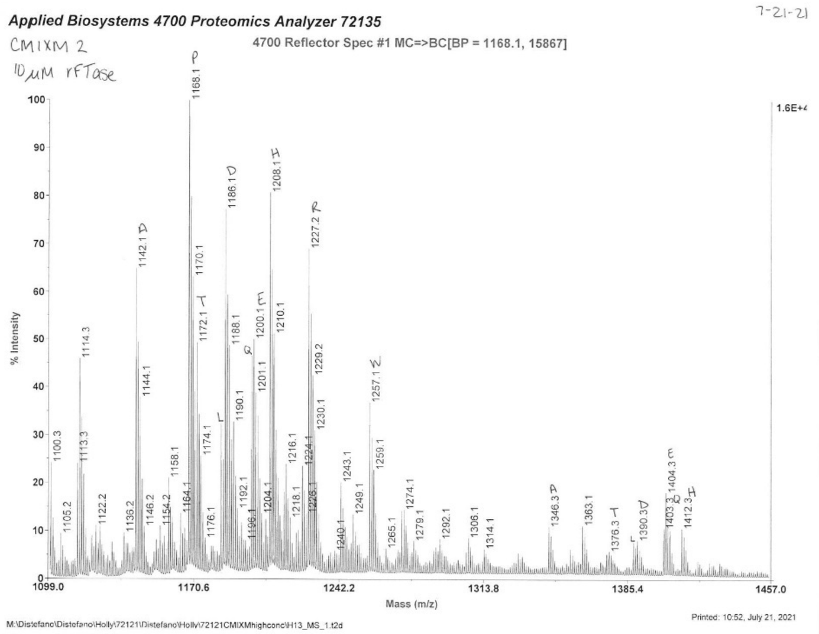

Figure S5. Analysis of CMIa$_2$M library 2 screened with rFTase before (above) and post reaction (below). The reaction was performed using 10 µM enzyme and analyzed by MALDI-MS.

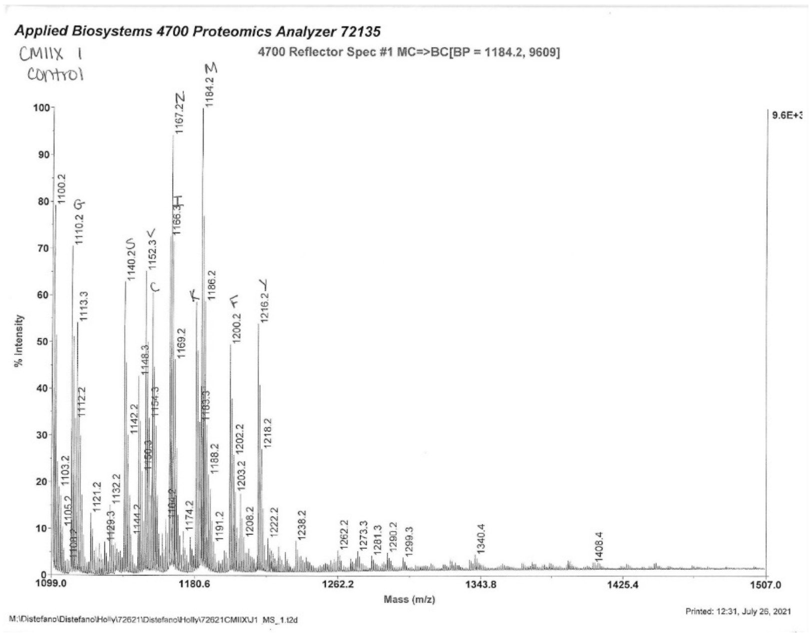

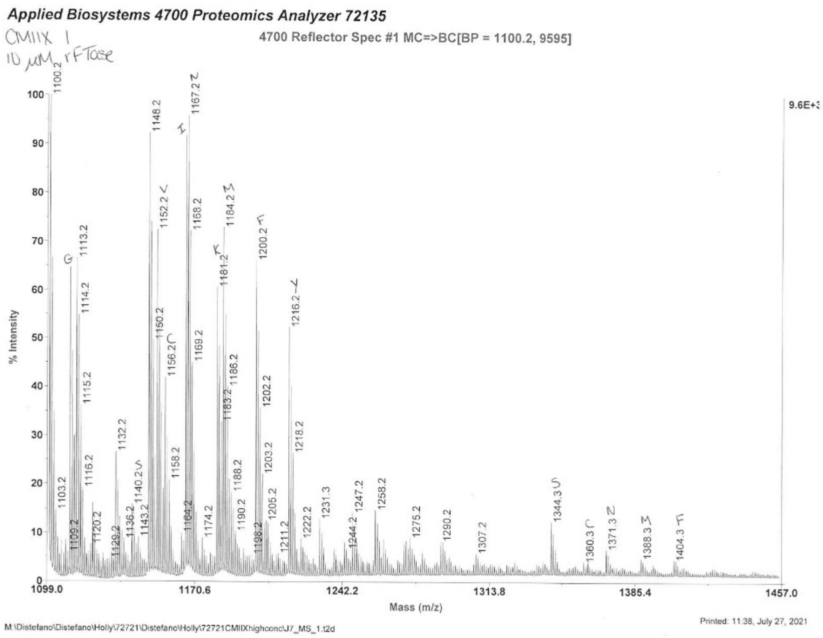

Figure S6. Analysis of CMIIX library 1 with rFTase before (above) and post reaction (below). The reaction was performed using 10 μM enzyme and analyzed by MALDI-MS.

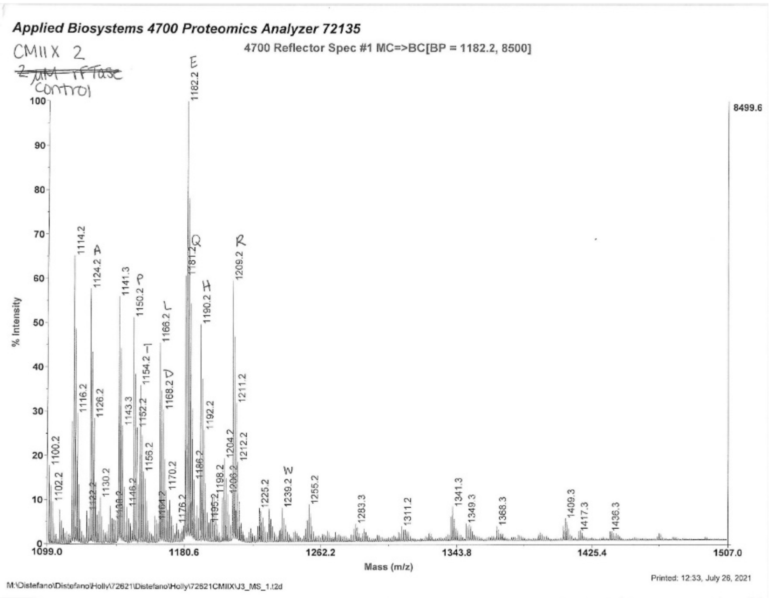

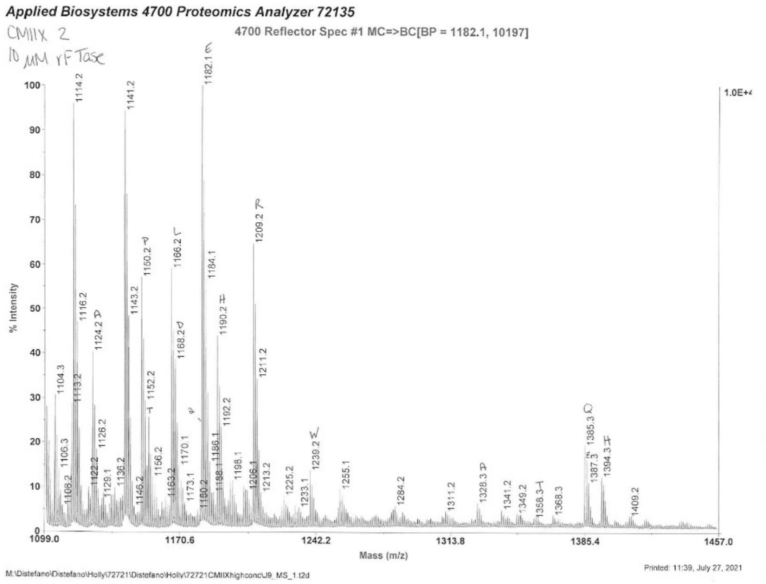

Figure S7. Analysis of the CMIIX library 2 with rFTase before (above) and post reaction (below). The reaction was performed using 10 μM enzyme and analyzed by MALDI-MS.

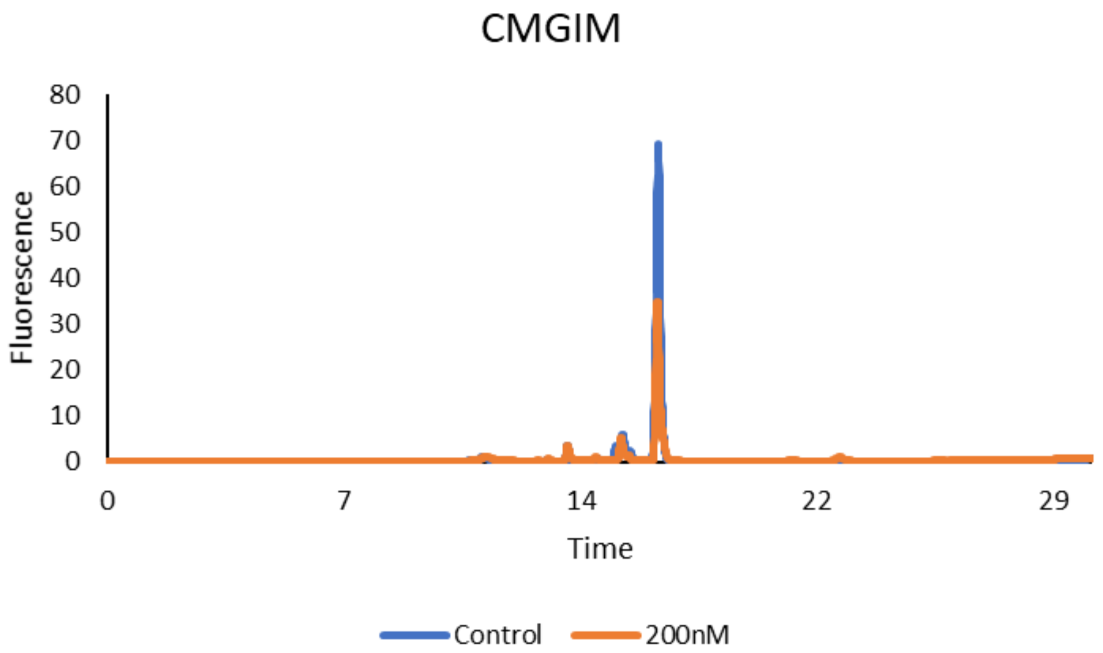

Figure S8. HPLC assay of CMGIM. Reaction contained 2.4 μM DsGRAGCMGIM with 200 nM rFTase for 45 min. Detection was accomplished by monitoring the fluorescence of the dansylated peptides using excitation at 220 nm and emission at 495 nm. The control reaction contained no enzyme.

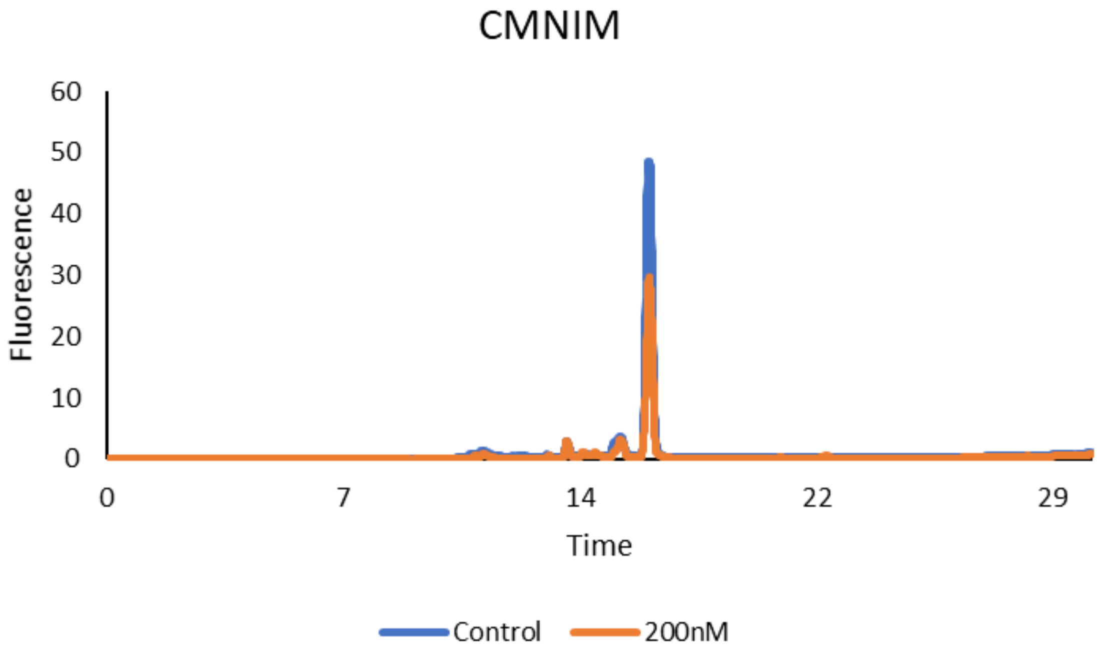

Figure S9. HPLC assay of CMNIM. Reaction contained 2.4 μM DsGRAGCMNIM with 200 nM rFTase for 45 min. Detection was accomplished by monitoring the fluorescence of the dansylated peptides using excitation at 220 nm and emission at 495 nm. The control reaction contained no enzyme.

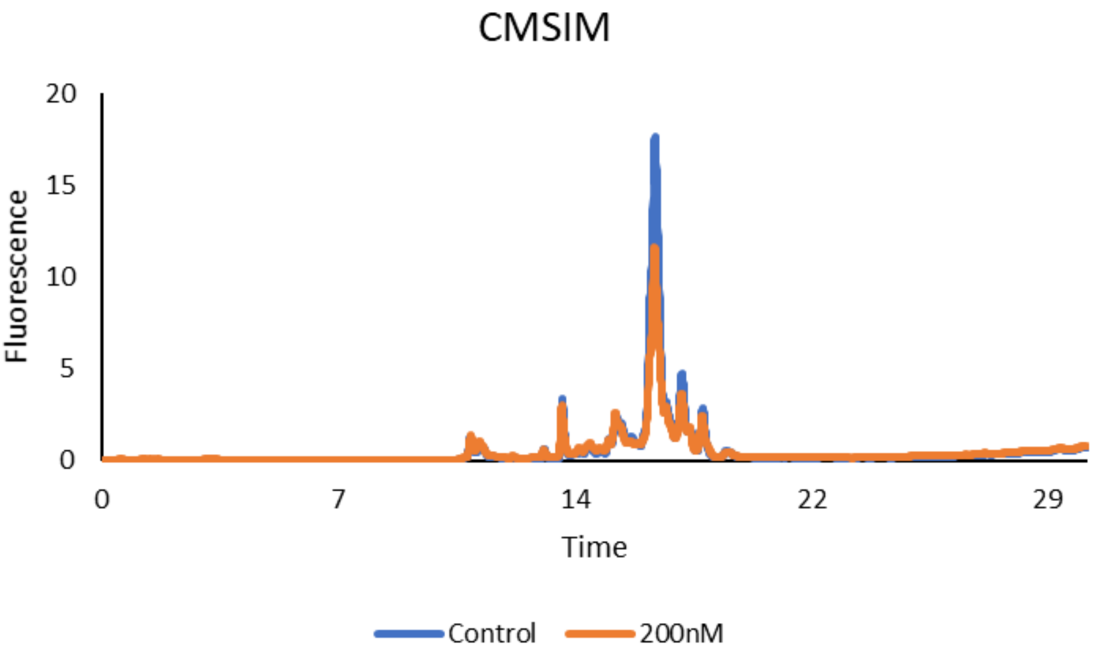

Figure S10. HPLC assay of CMSIM. Reaction contained 2.4 μM DsGRAGCMSIM with 200 nM rFTase for 45 min. Detection was accomplished by monitoring the fluorescence of the dansylated peptides using excitation at 220 nm and emission at 495 nm. The control reaction contained no enzyme.

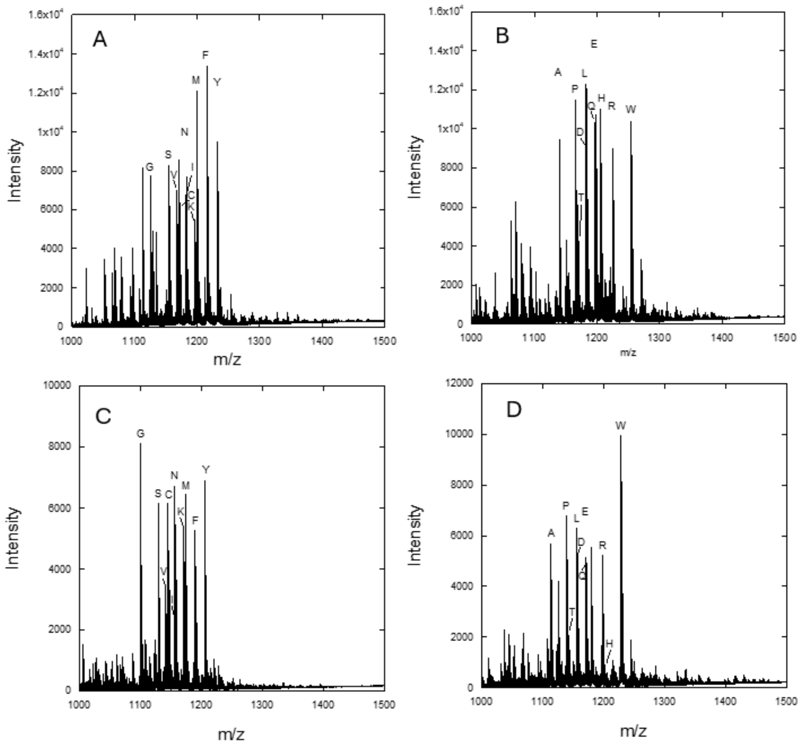

Figure S11. Part 1 (before reaction)
For legend, see next page.

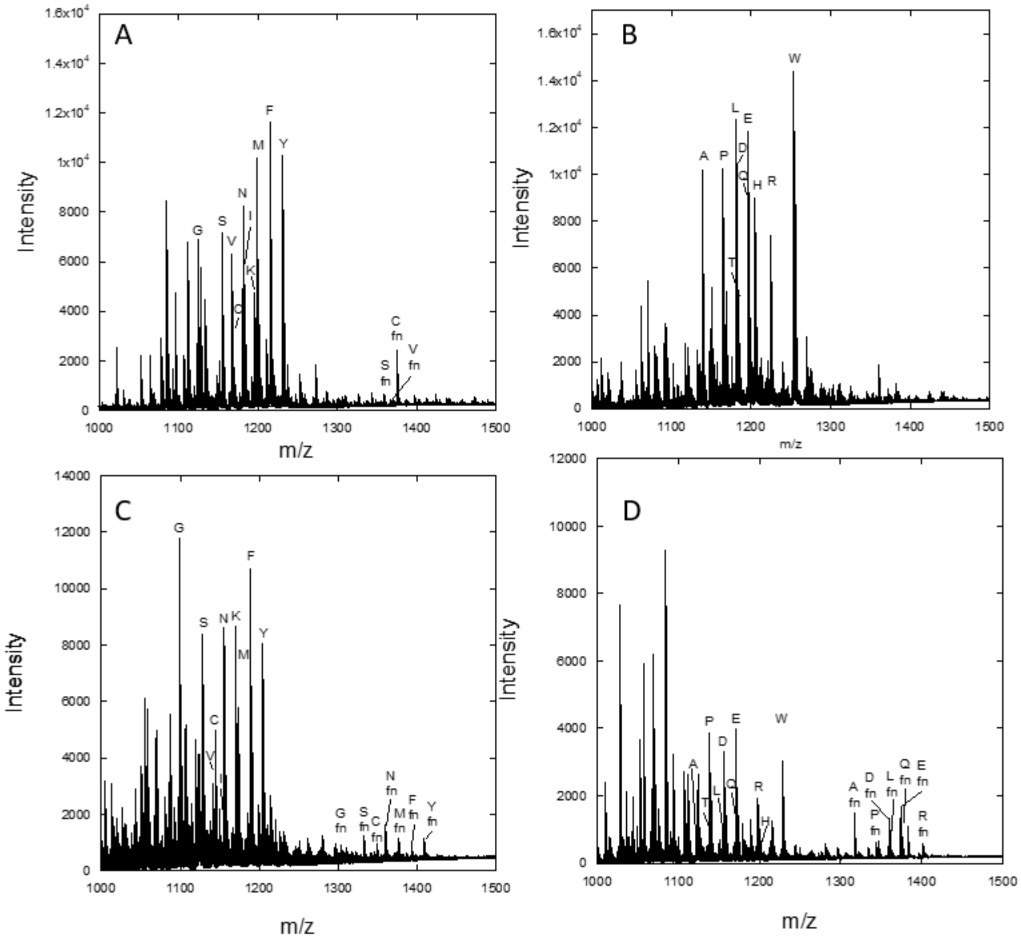

Figure S11. Part 2 (after reaction)
For legend, see below.

Figure S11. Analysis of CSLMQ a_0 and a_1 libraries screened with yFTase before (Part 1) and after (Part 2) enzymatic reaction. A. Ca_0LMQ library 1, B. Ca_0LMQ library 2, C. CSa_1MQ library 1, D. CSa_1MQ library 2. Reactions performed with 1 μM enzyme for 8 h and analyzed by MALDI-MS. “Fn” indicates a farnesylated product.

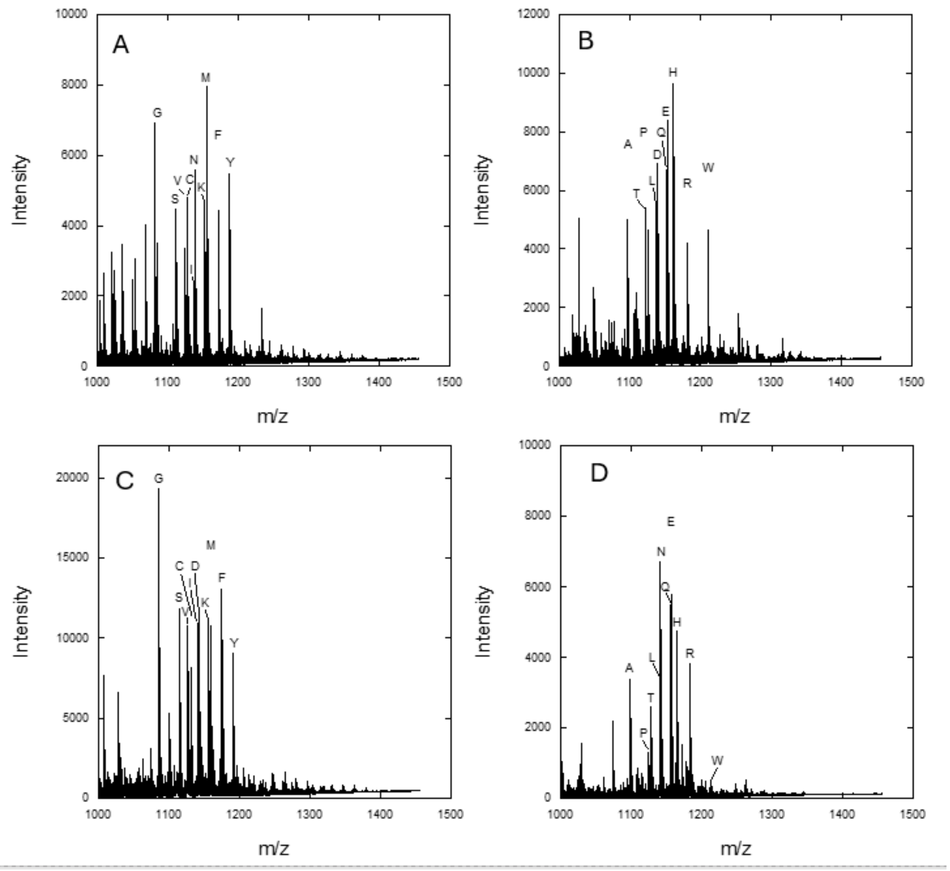

Figure S12. Part 1 (before reaction)
For legend, see next page.

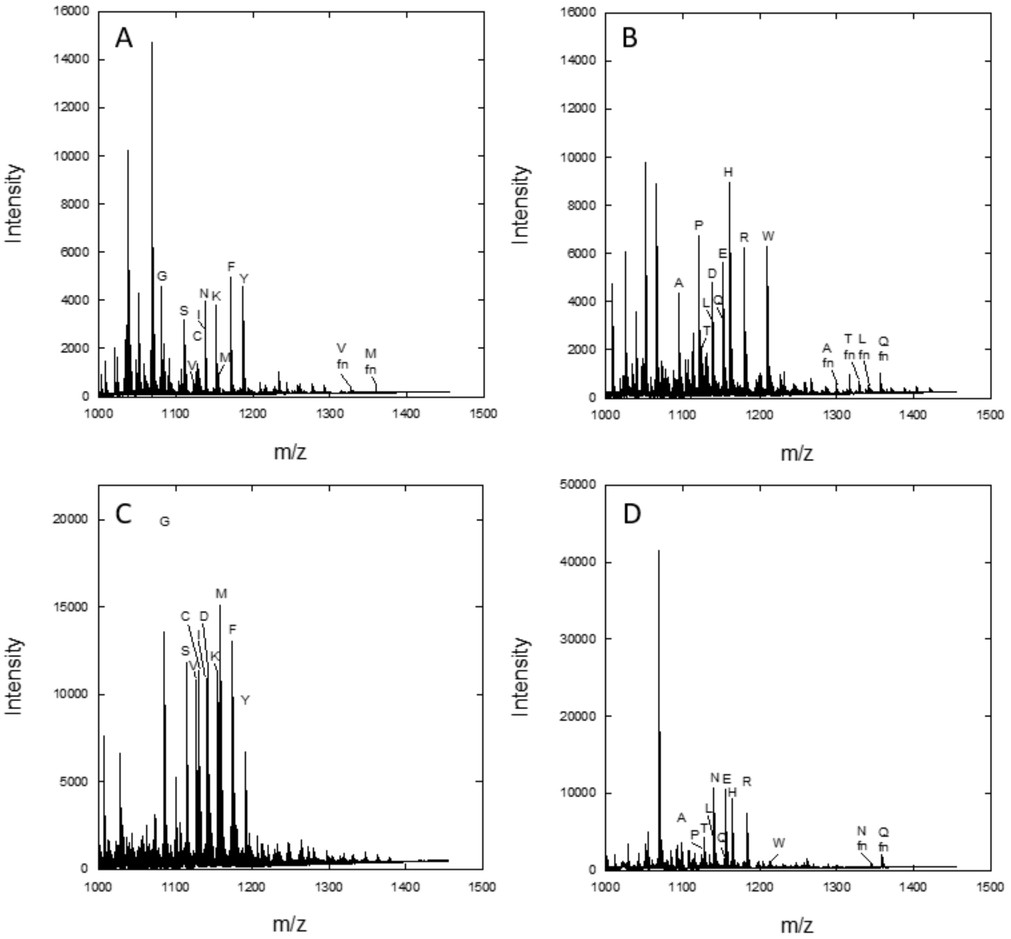

Figure S12. Part 2 (after reaction)
For legend, see below.

Figure S12. Analysis of CSLMQ a_2 and X libraries screened with yFTase before (Part 1) and after (Part 2) enzymatic reaction. A. $CSLa_2Q$ library 1, B. $CSLa_2Q$ library 2, C. CSLMX library 1, D. CSLMX library 2. Reactions performed with 1 µM enzyme for 8 h and analyzed by MALDI-MS. “Fn” indicates a farnesylated product.

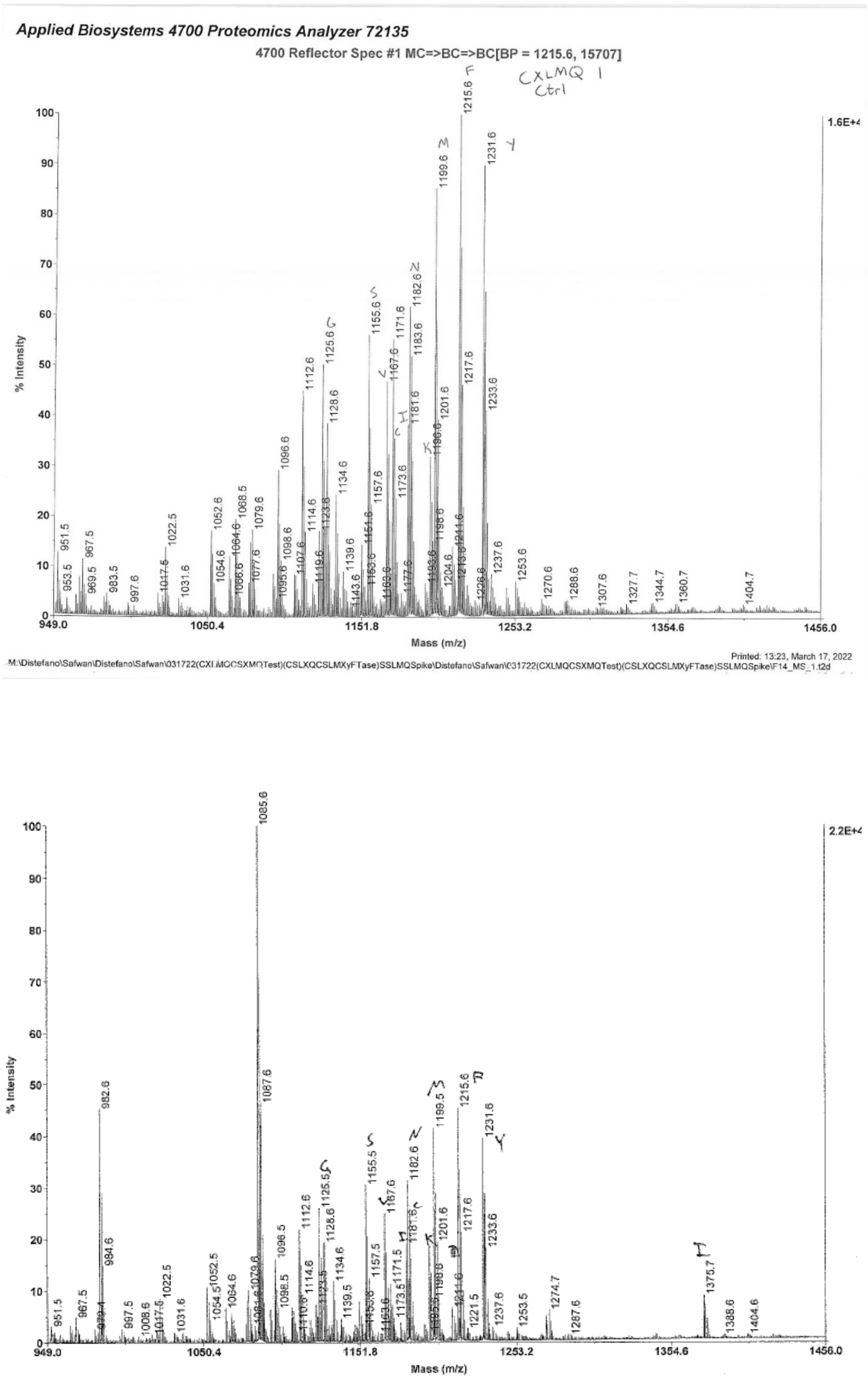

Figure S13. Analysis of Ca_0LMQ library 1 with screened rFTase. Reactions performed with 3 μM enzyme for 8 h and analyzed by MALDI-MS.

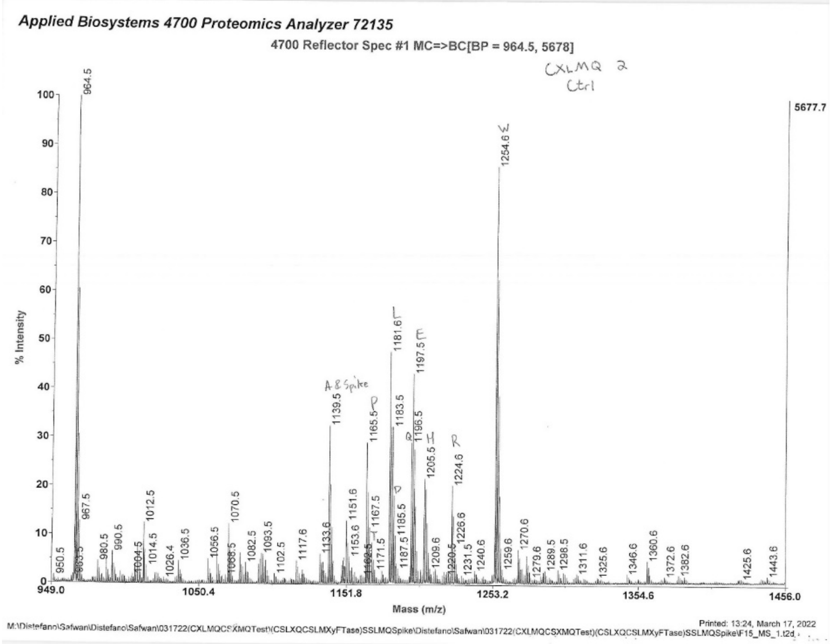

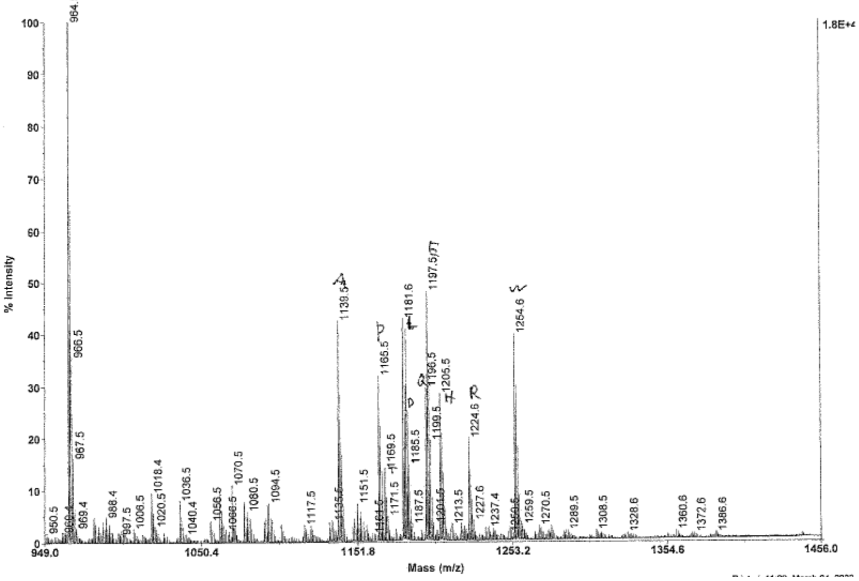

Figure S14. Analysis of Ca_0LMQ library 2 with rFTase. Reactions performed with 3 µM enzyme for 8 h and analyzed by MALDI-MS.

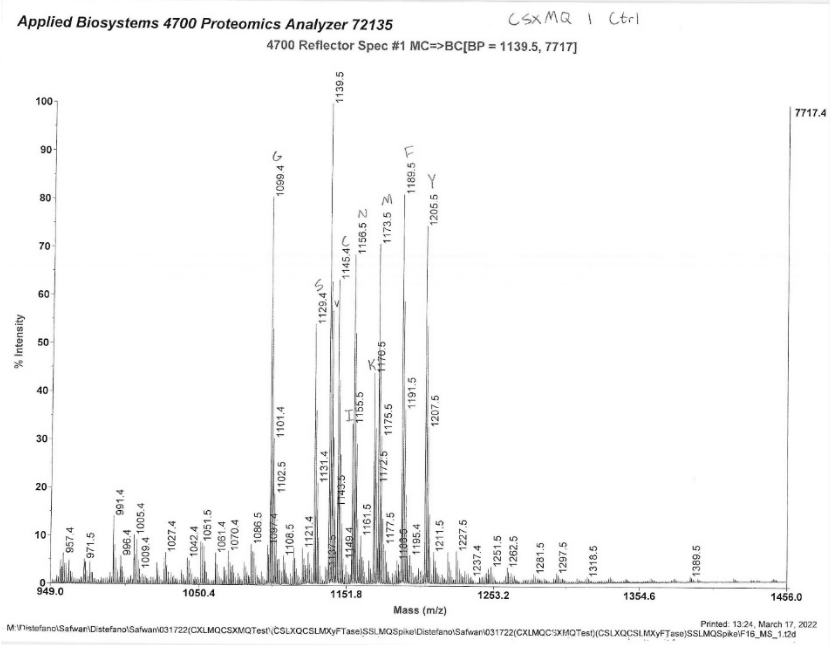

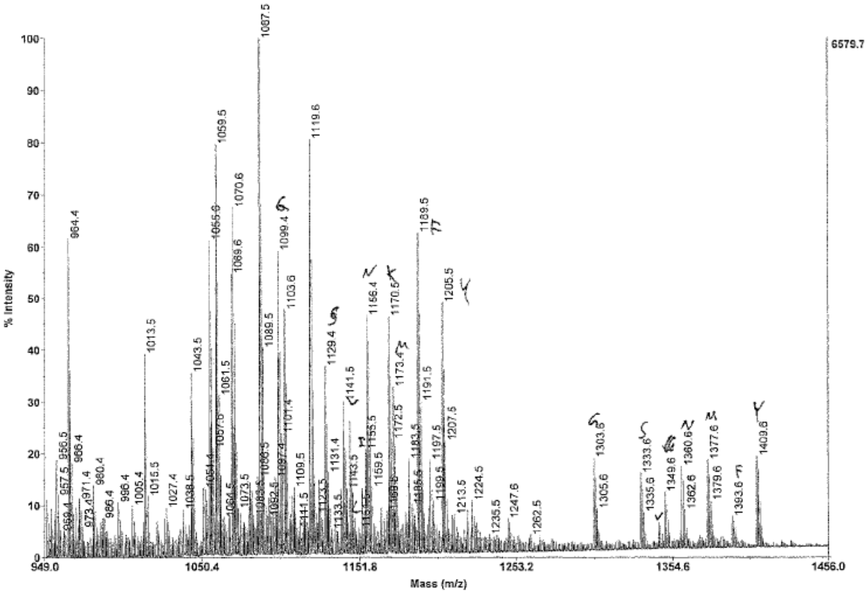

Figure S15. Analysis of CSa_1MQ library 1 screened with rFTase. Reactions performed with 3 μM enzyme for 8 h and analyzed by MALDI-MS.

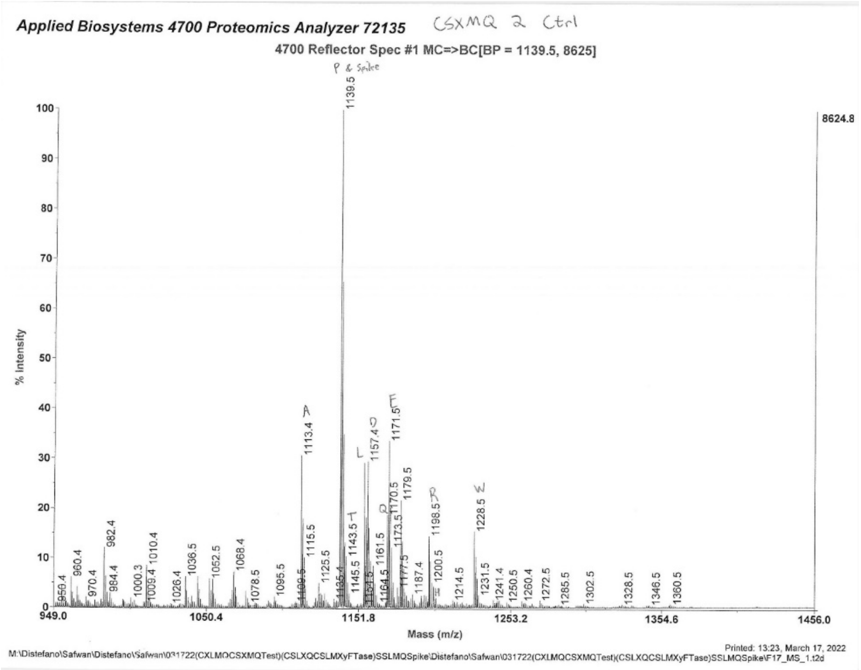

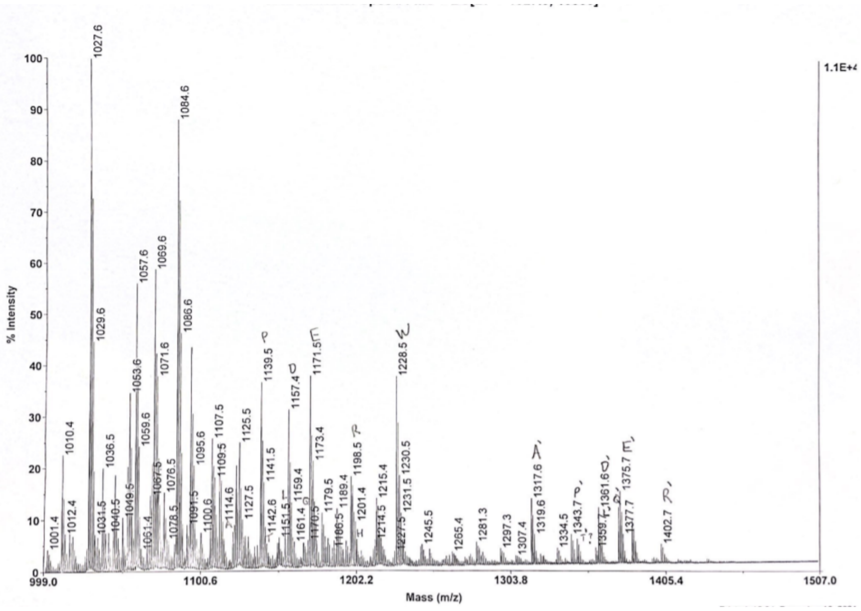

Figure S16. Analysis of CSa$_1$MQ library 2 screened with rFTase. Reactions performed with 3 μM enzyme for 8 h and analyzed by MALDI-MS.

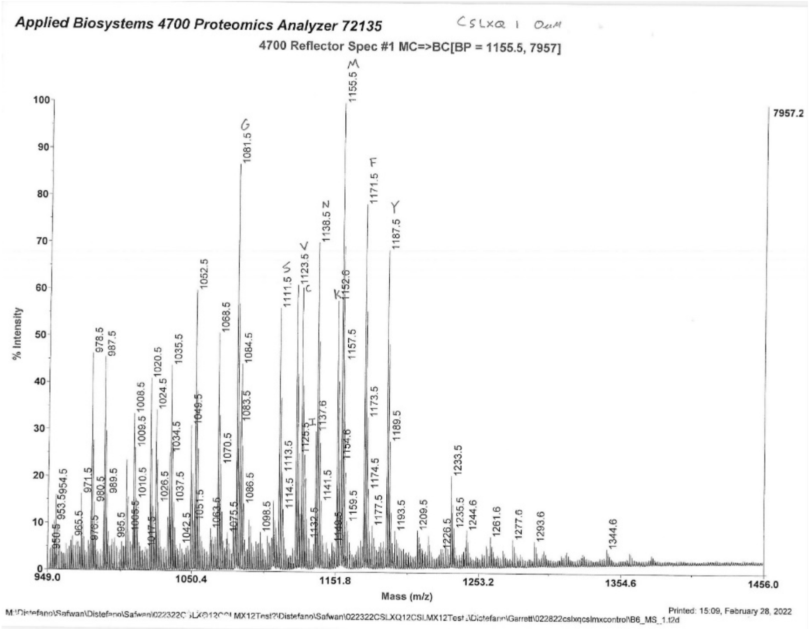

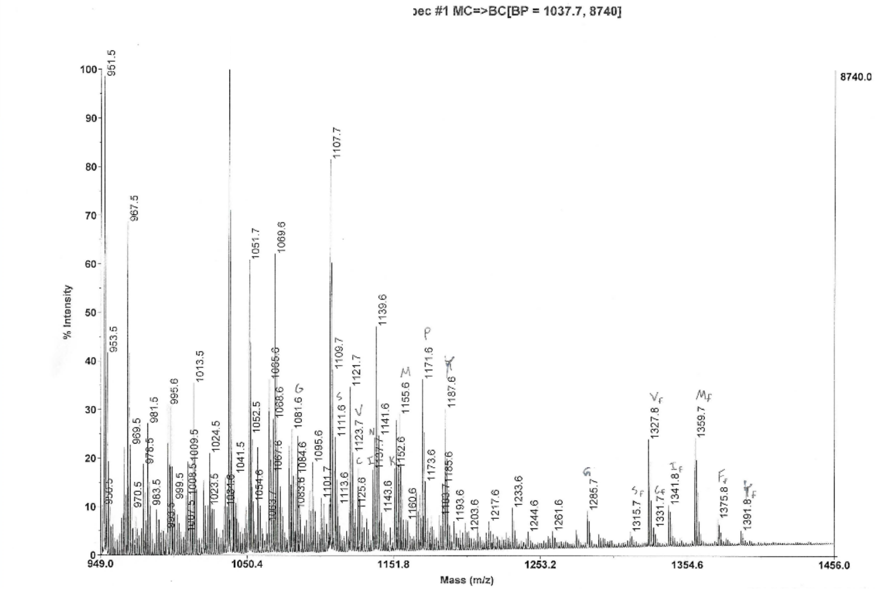

Figure S17. Analysis of CSLa$_2$Q library 1 screened with rFTase. Reactions were performed with 3 μM enzyme for 8 h and analyzed by MALDI-MS.

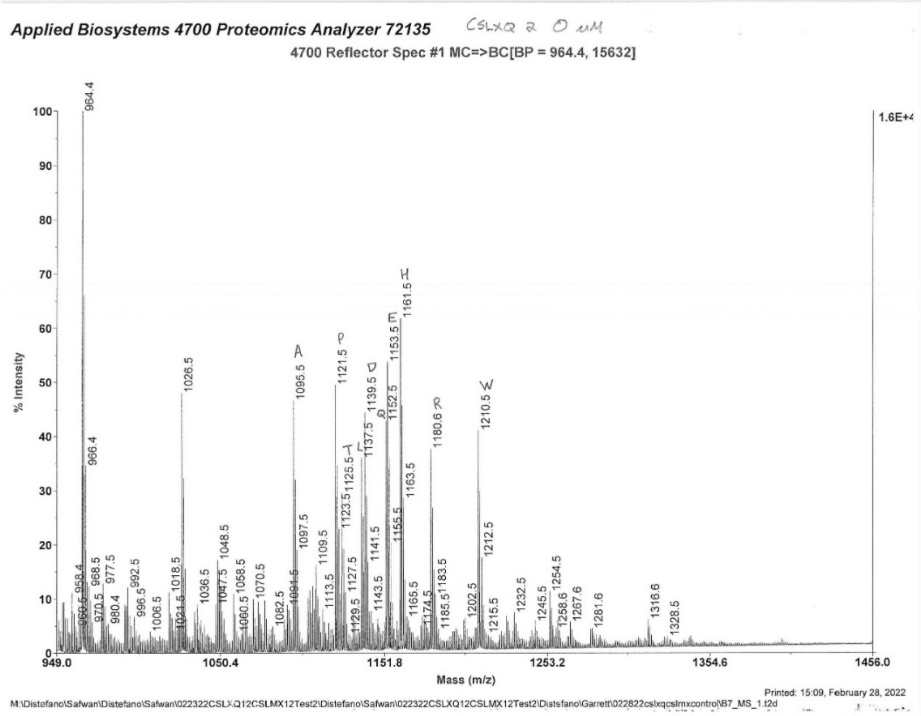

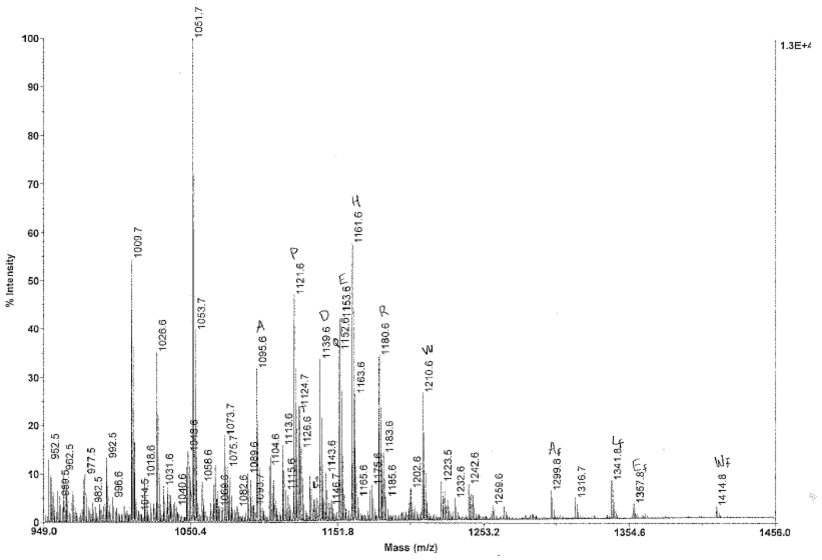

Figure S18. Analysis of $CSLa_2Q$ library 2 screened with rFTase. Reactions were performed with 3 μM enzyme for 8 h and analyzed by MALDI-MS.

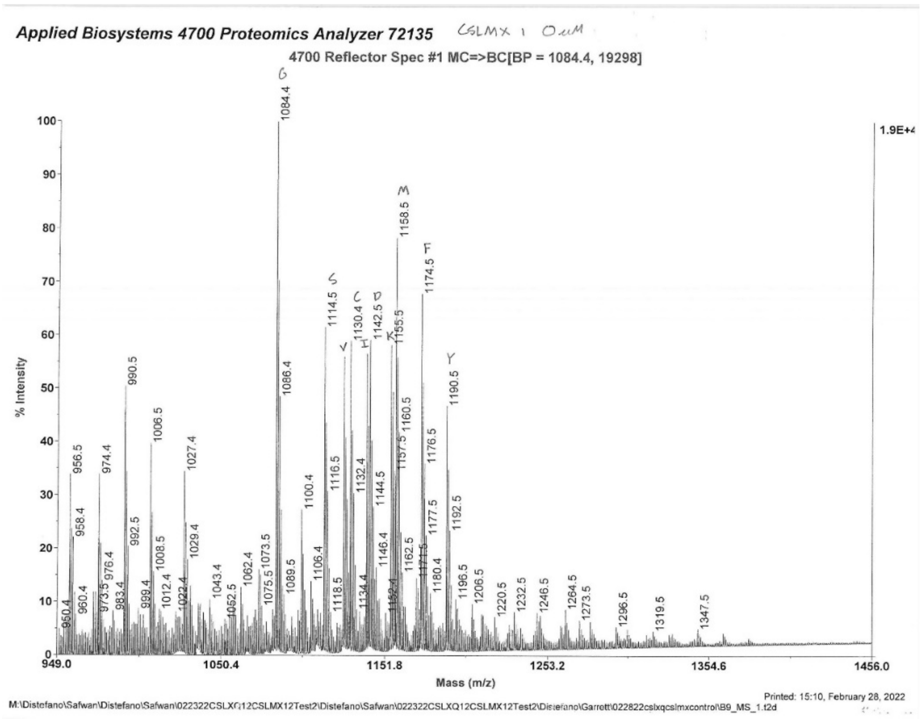

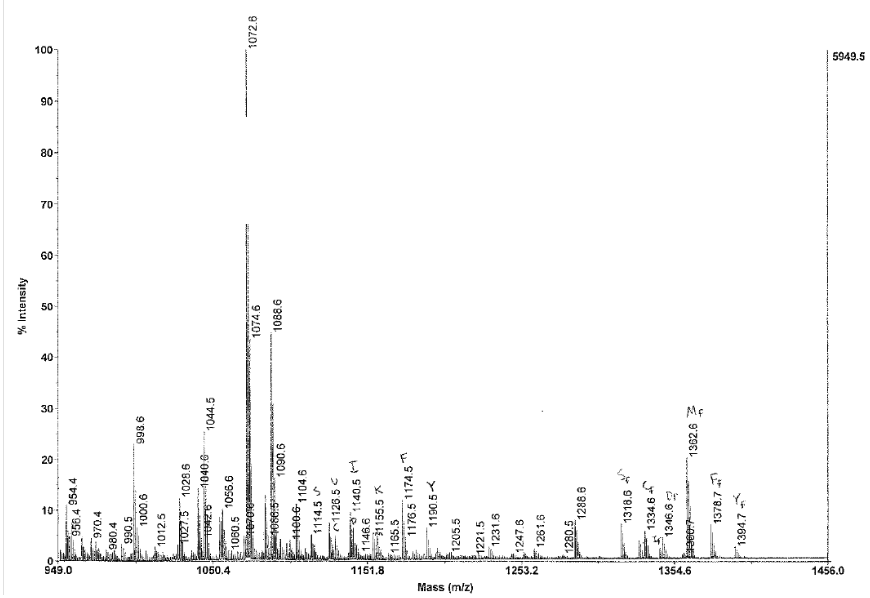

Figure S19. Analysis of CSLMX library 1 screened with rFTase. Reactions were performed with 3 µM enzyme for 8 h and analyzed by MALDI-MS.

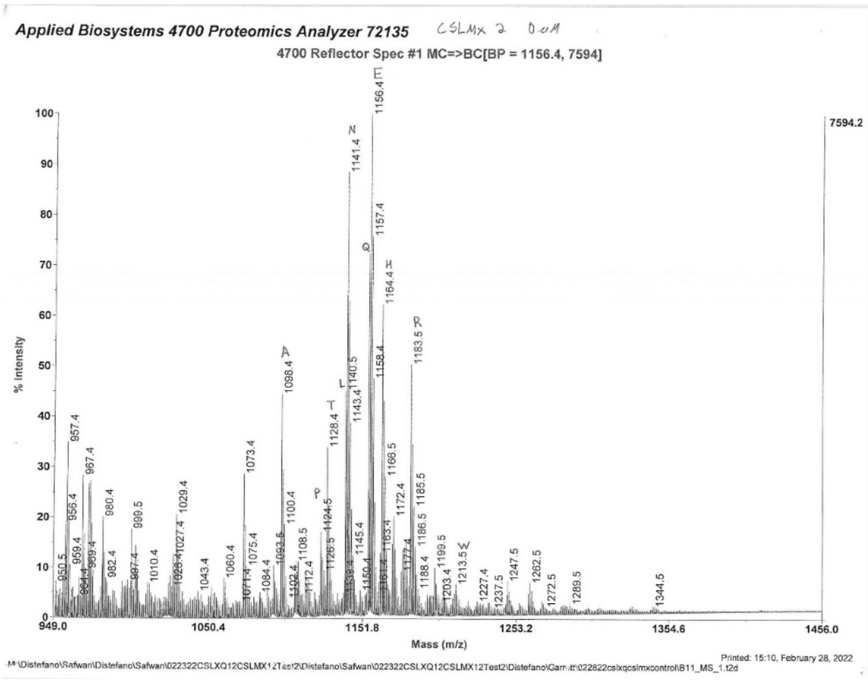

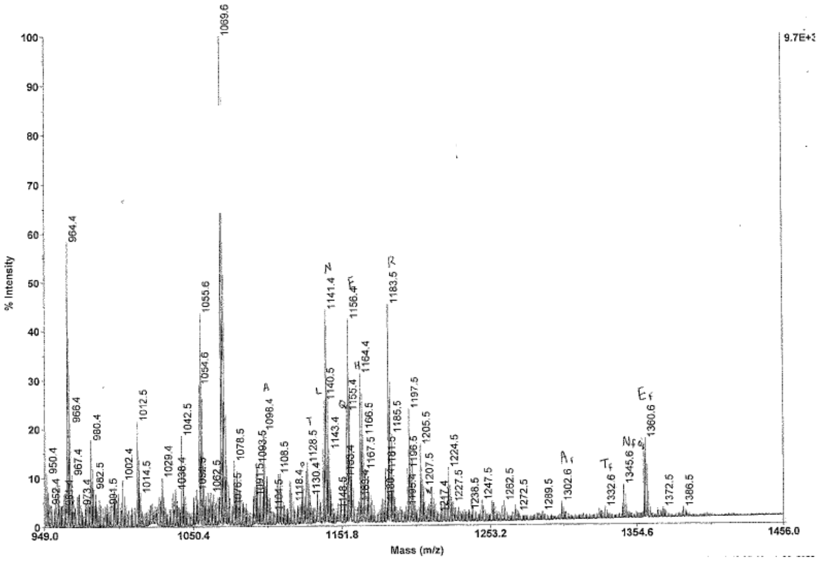

Figure S20. Analysis of CSLMX library 2 screened with rFTase. Reactions were performed with 3 μM enzyme for 8 h and analyzed by MALDI-MS.

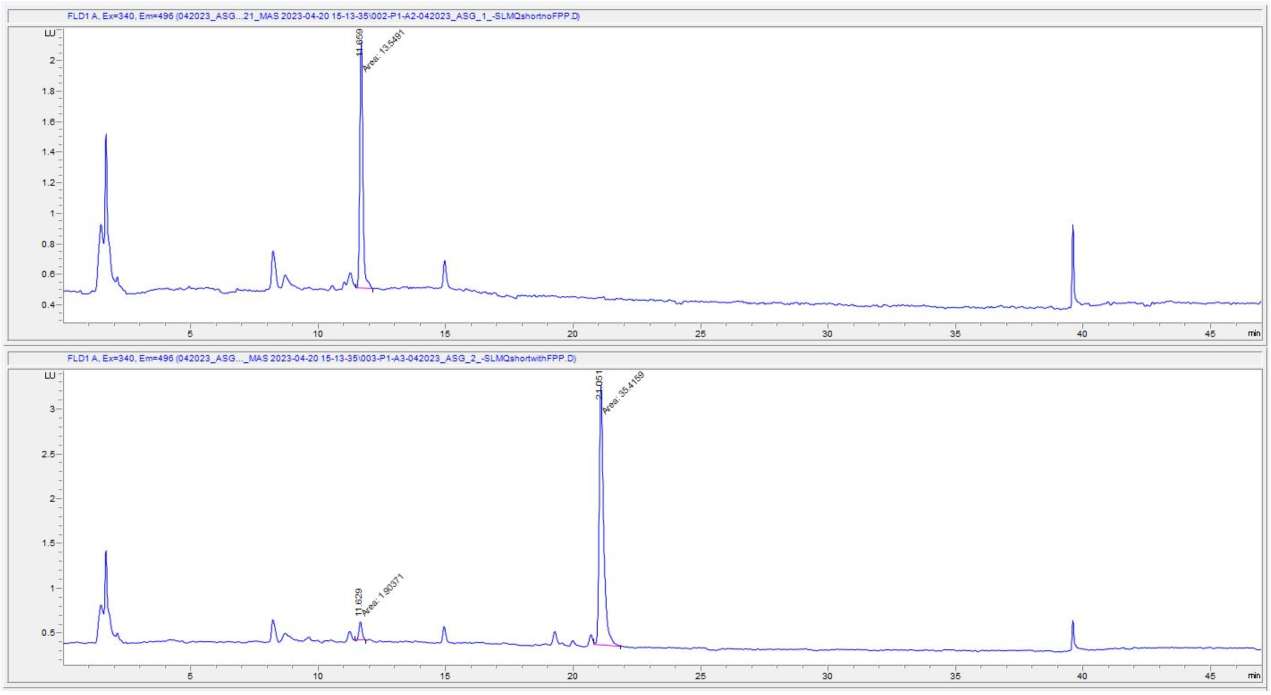

Figure S21. HPLC assay of CSLMQ. Reaction contained 3 µM DsGCSLMQ before (top) and after (bottom) addition of 100 nM rFTase. Detection was accomplished by monitoring the fluorescence of the dansylated peptide using excitation at 340 nm and emission at 496 nm. Note: The improved S/N ratio obtained via excitation at 220 nm instead of 340 nm is readily apparent when comparing this data with Figure S22. This is why subsequent experiments employed 220 nm excitation.

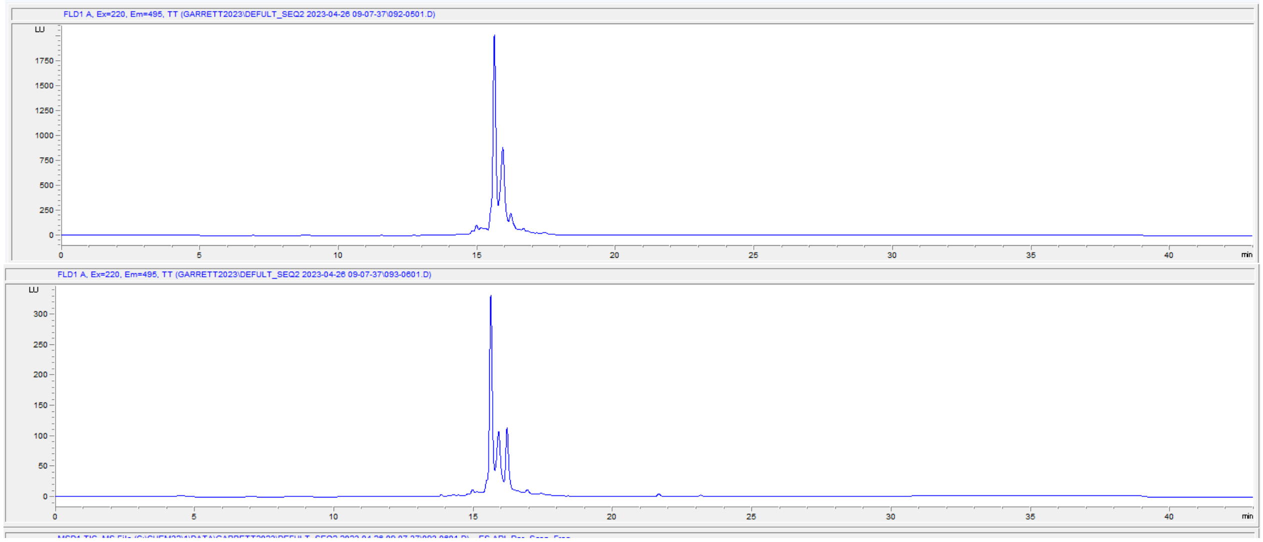

Figure S22. HPLC assay of CMSIM. Reaction contained 2.4 µM DsGCMSIM before (top) and after (bottom) addition of 100 nM yFTase. While there is a large decrease in the starting material, the (apparently) insoluble product is not observed. Precipitation of starting material was not observed. Detection was accomplished by monitoring the fluorescence of the dansylated peptide using excitation at 220 nm and emission at 495 nm.

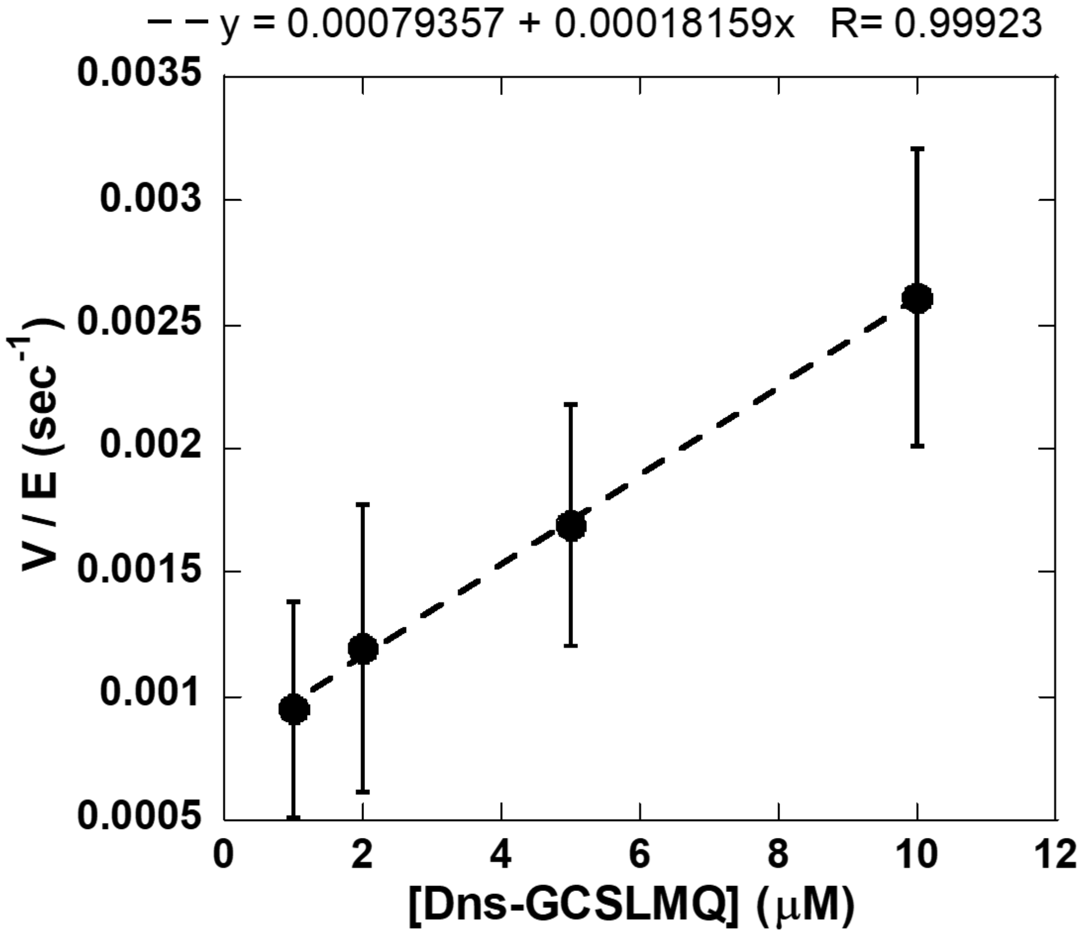

Figure S23. Kinetic analysis of CSLMQ prenylation by rat FTase. The initial velocity for modification of the peptide substrate Dansyl-GCSLMQ was determined at varying concentrations of the peptide substrate. For the plot, the initial velocity (rate) was divided by the enzyme concentration and ploted versus peptide concentration. Linear regression analysis gave a line whose slope approximates k_{cat}/K_M (when [S] < K_M). A value of k_{cat}/K_M for Dansyl-GCSLMQ using rFTase was determined although poor peptide binding (nonsaturating behavior) precluded measurement of distinct k_{cat} and K_M values. This analysis gave a value of k_{cat}/K_M of 182 $sec^{-1}M^{-1}$.

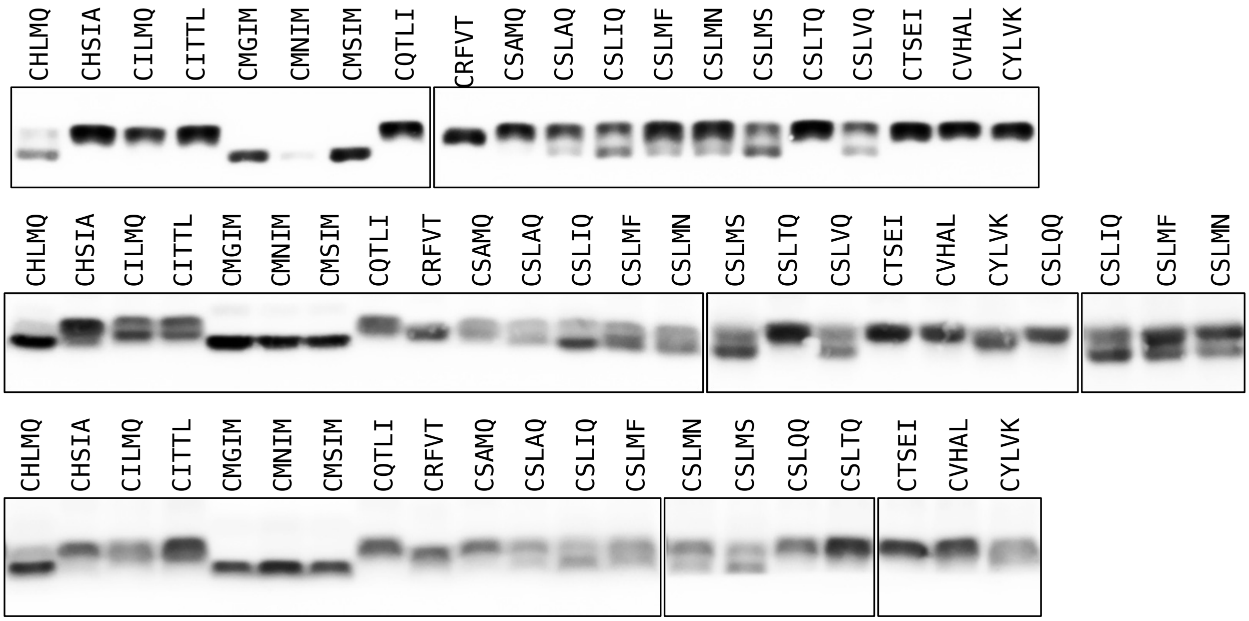

Figure S24. Western blot analysis of Ydj1-CaaaX samples. Each iteration of a specific CaaaX sequence represents either a technical or biological replicate. The intensities of the upper (unfarnesylated) and lower (farnesylated) bands were determined for each sample and used to calculate the percent farnesylation of a sequence set, which is reported in **Table S2**.

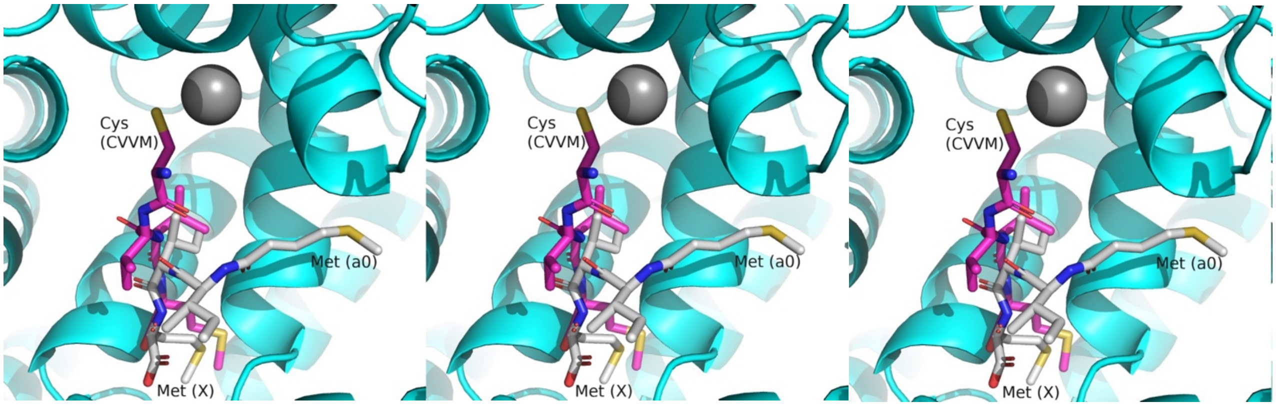

Figure S25. Alternative stereo image version of Figure 4. Stereo image of superposition of previously solved structure of the peptide CVVM (pink carbons) bound to CnFTase (cyan, pdb id 3q75) aligned with the newly reported structure of CMIIM (white carbons) bound to CnFTase (omitted for clarity, pdb id 8t70). Peptide atoms: N (blue); O (red); S (yellow); Zn (grey).The Cys, a_0, and X positions in the $Ca_0a_1a_2X$ box are labeled. Using the Left and Center images, the cross-eyed stereo image can be viewed. Using the Center and Right images, the parallel stereo image can be viewed.

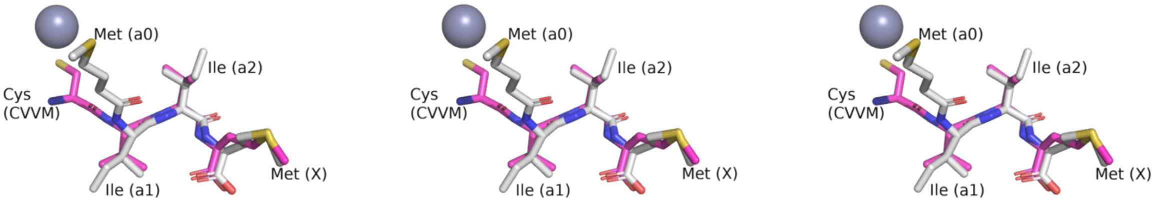

Figure S26. Stereoview of superposition of TKCVVM and CMIIM bound to CnFTase. CVVM is shown in magenta and CMIIM is shown in white. There is clear overlap between the C-terminal tripeptide. Cys residues are labeled, as are the a_0 a_1, a_2 and X residues in the $Ca_0a_1a_2X$ box. Using the Left and Center images, the cross-eyed stereo image can be viewed. Using the Center and Right images, the parallel stereo image can be viewed.

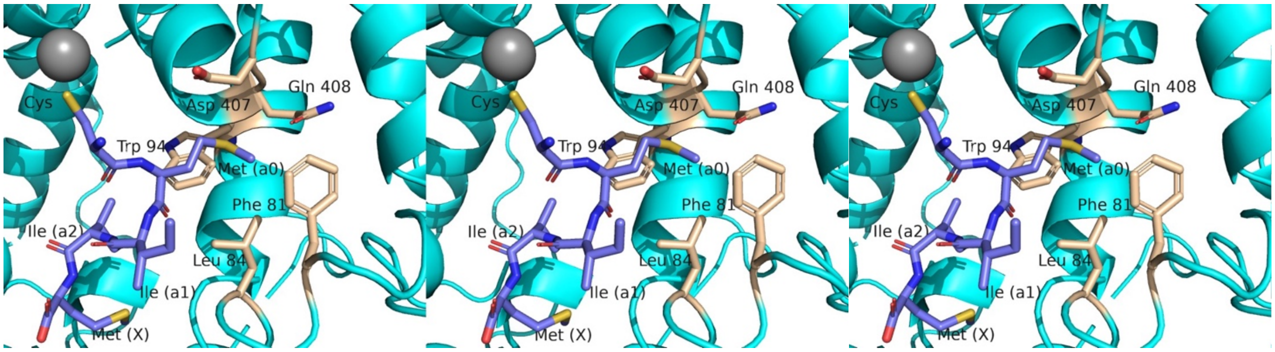

Figure S27. Alternative stereo image version of Figure 5. Stereo image of modeling of X-ray structure of CMIIM, modeling Cys to chelate Zn followed by molecular dynamics.. The residues surrounding the a_0 Met are shown in tan sticks and labeled. Peptide atoms: N (blue); O (red); S (yellow); Zn (grey). The Cys, a_0, a_1, and X positions in the $Ca_0a_1a_2X$ box are labeled. Using the Left and Center images, the cross-eyed stereo image can be viewed. Using the Center and Right images, the parallel stereo image can be viewed.

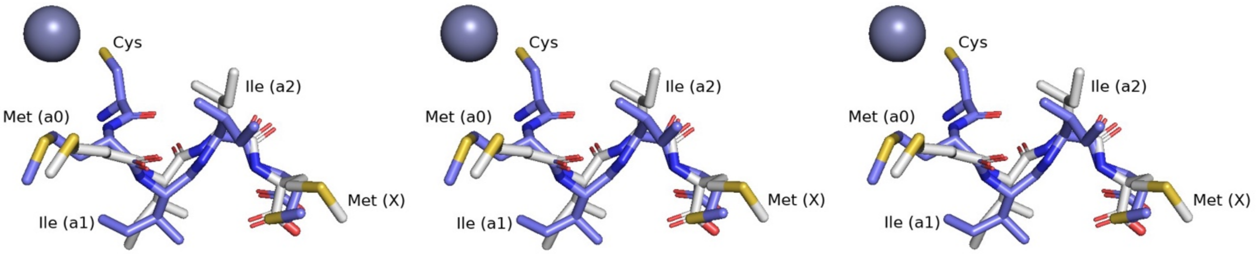

Figure S28. Stereoview of superposition crystal structure of CMIIM before and after modeling and molecular dynamics of the Cys to coordinate the active site Zn. CMIIM is shown in white and after molecular dynamics in blue. Some limited movement of the peptide was observed . Cys residues, a_0, a_1, a_2 and X residues in the $Ca_0a_1a_2X$ box are labeled. Using the Left and Center images, the cross-eyed stereo image can be viewed. Using the Center and Right images, the parallel stereo image can be viewed.

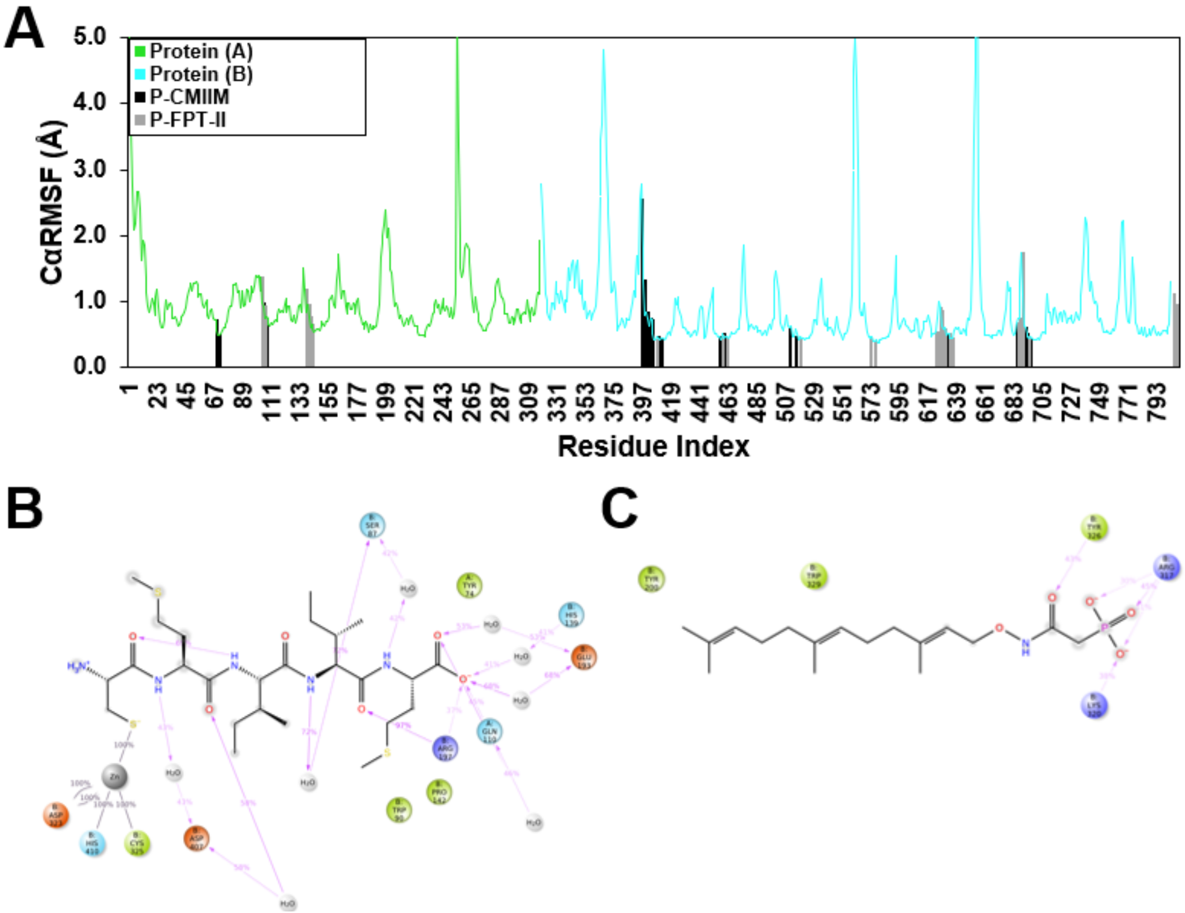

Figure S29. MD simulation of CMIIM bound to CnFTase. (A) C_αRMSD plot of CnFTase over the course of the 200 ns MD simulation. Ligand interaction plot for CMIIM (B) and FPT-II (C) within the CnFTase active site.

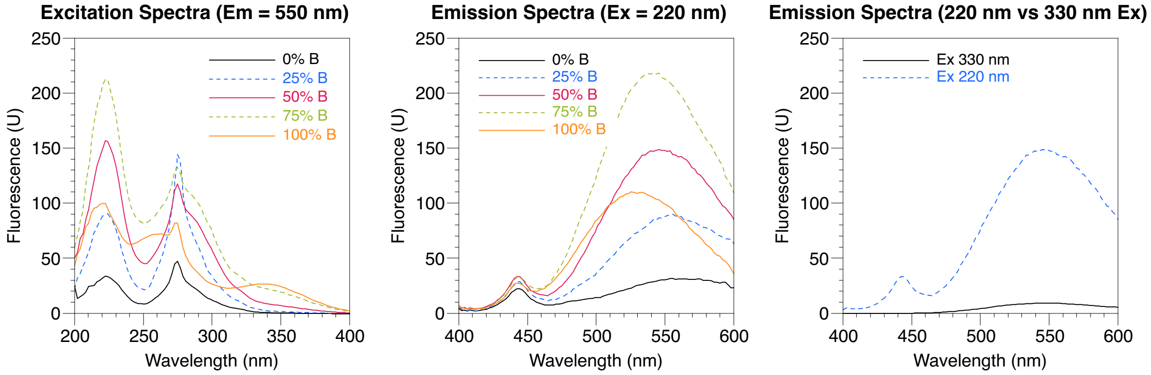

Figure S30. Fluorescence spectra for Dansyl-Gly in different solvent mixtures. (Left): Excitation spectra of Dansyl-Gly observed via fluorescence detection at 550 nm obtained in different mixtures of Buffer A and Buffer B including 0%, 25%, 50%, 75% and 100% Buffer B. (Center): Emission spectra of Dansyl-Gly observed by excitation at 220 nm obtained in different mixtures of Buffer A and Buffer B including 0%, 25%, 50%, 75% and 100% Buffer B. (Right): Emission spectra of Dansyl-Gly observed by excitation at 220 nm or 330 nm. All spectra were acquired using 9.6 µM Dansyl-Gly. Buffer A: H_2O/0.1% TFA; Buffer B: CH_3CN/0.1% TFA.

Table S1. List of 192 CaaaX sequences synthesized from the human genome and their calculated PrePS and Ras HM scores based on the three C-terminal residues.

This Table is included as a separate Excel file.

Table S2. Summary of gel-shift data obtained from Western blotting of extracts obtained by expression of CaaaX-box sequences fused to the C-terminus of Ydj1. Variants averaging farnesylation below 20% were considered unmodified.

CaaaX seq.	% farn.	SEM[1]	n	95% Confidence Interval	Why chosen?
CM**G**IM	100	0	5	0	CM**a_1**IM library hit
CM**N**IM	100	0	5	0	CM**a_1**IM library hit
CM**S**IM	100	0	5	0	CM**a_1**IM library hit
C**H**LMQ	80.8	3.1	5	2.9	C**a_0**LMQ library hit[2]
C**I**LMQ	17.3	11.1	5	10.5	C**a_0**LMQ library hit
CS**A**MQ	18.7	10.6	5	10.1	CS**a_1**MQ library hit
CSL**I**Q	59.6	4.4	7	4.2	CSL**a_2**Q library hit
CSL**V**Q	44.2	7.0	4	6.7	CSL**a_2**Q library hit
CSL**A**Q	34.7	6.3	6	6.0	CSL**a_2**Q library hit
CSL**T**Q	0	0	6	0	CSL**a_2**Q library hit
CSLM**S**	58.7	3.7	5	3.5	CSLM**X** library hit
CSLM**F**	38.2	8.0	6	7.6	CSLM**X** library hit
CSLM**N**	36.0	6.6	6	6.3	CSLM**X** library hit
CHSIA	14.4	2.6	7	2.5	genome
CITTL	11.0	9.1	5	8.6	genome
CQTLI	8.5	6.7	5	6.4	genome
CYLVK	7.0	7.0	5	6.7	genome
CRFVT	0	0	5	0	genome
CTSEI	0	0	5	0	genome
CVHAL	0	0	6	0	genome
CSL**Q**Q	0	0	4	0	genome[3]
C**H**IIM	94.2	1.9	7	1.8	previous work[4]

[1]SEM – standard error of the mean.
[2]While not a hit in our *in vitro* library analysis, this peptide was chosen for *in vivo* analysis since His in the a0 position has previously been shown to be well prenylated in *in vivo* assays.
[3]While not observed as a hit in our *in vitro* library analysis (although the unprenylated peptide was present in the library) using rFTase, it was observed using yFTase. This peptide was chosen for *in vivo* analysis since that sequence was found to occur in the human genome.
[4]This sequence was identified in previous work (Schey *et al.*, *Int. J. Mol. Sci.* **2021**, *22*, 12042).

Table S3. Plasmids used in these studies.

identifier	genotype	source
pWS1132	*CEN URA3 YDJ1-SASQ*	Hildebrandt et al, eLife
pWS2259	*CEN URA3 YDJ1-CHLMQ*	This study
pWS2260	*CEN URA3 YDJ1-CHSIA*	This study
pWS2261	*CEN URA3 YDJ1-CILMQ*	This study
pWS2262	*CEN URA3 YDJ1-CITTL*	This study
pWS2263	*CEN URA3 YDJ1-CMGIM*	This study
pWS2264	*CEN URA3 YDJ1-CMNIM*	This study
pWS2265	*CEN URA3 YDJ1-CMSIM*	This study
pWS2266	*CEN URA3 YDJ1-CQTLI*	This study
pWS2267	*CEN URA3 YDJ1-CRFVT*	This study
pWS2268	*CEN URA3 YDJ1-CSAMQ*	This study
pWS2269	*CEN URA3 YDJ1-CSLAQ*	This study
pWS2270	*CEN URA3 YDJ1-CSLIQ*	This study
pWS2271	*CEN URA3 YDJ1-CSLMF*	This study
pWS2272	*CEN URA3 YDJ1-CSLMN*	This study
pWS2273	*CEN URA3 YDJ1-CSLMS*	This study
pWS2274	*CEN URA3 YDJ1-CSLTQ*	This study
pWS2275	*CEN URA3 YDJ1-CSLVQ*	This study
pWS2276	*CEN URA3 YDJ1-CTSEI*	This study
pWS2277	*CEN URA3 YDJ1-CVHAL*	This study
pWS2278	*CEN URA3 YDJ1-CYLVK*	This study

Table S4. Summary of data collection and refinement information for the cystal structure of TKCMIIM and FII bound to CnFTase.

**Data collection**	
Wavelength	1.000
Resolution range	49.77 - 1.89 (1.96 - 1.89)
Multiplicity	5.7 (5.9)
Completeness (%)	99.25 (98.87)
Mean I/sigma(I)	8.08 (1.82)
R_{merge}	0.117 (0.6878)
R_{pim}	0.05334 (0.3061)
**Refinement**	
Reflections used in refinement	102952 (10129)
R_{work}	0.1849 (0.2752)
R_{free}	0.2118 (0.3208)
Number of non-hydrogen atoms	6964
macromolecules	6423
solvent	372
Protein residues	808
RMS(bonds)	0.006
RMS(angles)	0.80
Ramachandran favored (%)	97.74
Ramachandran allowed (%)	2.26
Ramachandran outliers (%)	0.00
Rotamer outliers (%)	1.00
Average B-factor	36.91
Protein	36.65
TKCMIIM peptide	43.06
FPT-II	38.63

Table S5. List and bioinformatic analysis of all CaaaX-box sequences selected for further study after MALDI screening. All of these sequences were identified as positive hits via MALDI screening. This list also includes positives from a previous study. This data was used to generate the graphs shown in Figure 6.

This Table is included as a separate Excel file.

Table S6. List of all amino acids used in each library, as well as the prenylated hits observed.

This Table is included as a separate Excel file.

Supplemental Movie File.

This movie is included as a separate (.mpeg) file.
